# pH-Responsive Semi-Interpenetrated Polymer Networks of pHEMA/PAA for the Capture of Copper Ions and Corrosion Removal

**DOI:** 10.1021/acsami.1c22837

**Published:** 2022-01-28

**Authors:** Teresa Guaragnone, Marta Rossi, David Chelazzi, Rosangela Mastrangelo, Mirko Severi, Emiliano Fratini, Piero Baglioni

**Affiliations:** Department of Chemistry “Ugo Schiff” and CSGI, University of Florence, via della Lastruccia 3-Sesto Fiorentino, I-50019 Florence, Italy

**Keywords:** semi-IPN hydrogels, pHEMA, PAA, PVP, TEPA, copper ions, cleaning bronze, corrosion removal, Cultural Heritage conservation

## Abstract

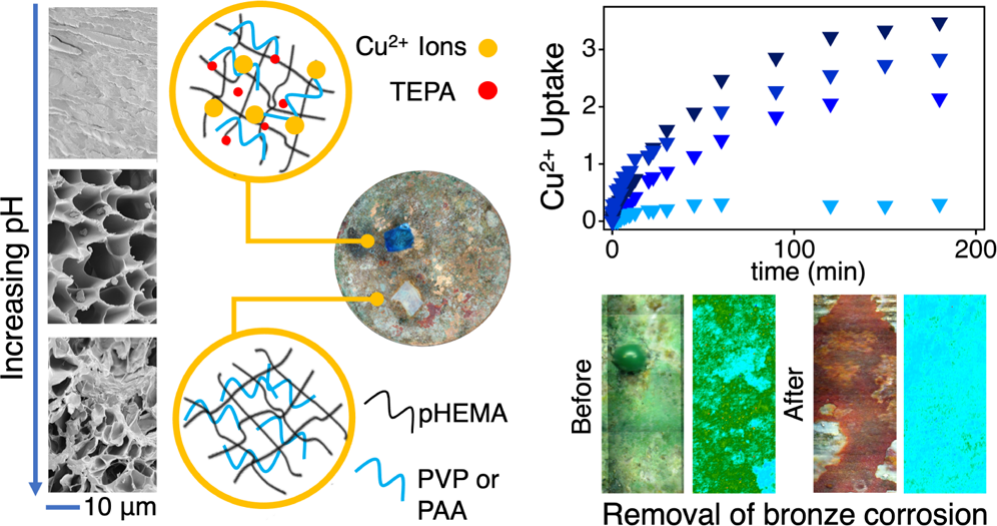

Bronze artifacts constitute a fundamental portion of Cultural Heritage, but effective methodologies for the removal of corrosion layers, such as those produced by the “bronze disease”, are currently missing. We propose the formulation and application of novel poly(2-hydroxyethyl methacrylate) (pHEMA) networks semi-interpenetrated (SIPN) with poly(acrylic acid) (PAA) to achieve enhanced capture of copper ions and removal of corrosion products. The pHEMA/PAA SIPNs were designed to improve previous pHEMA/poly(vinylpyrrolidone) (PVP) networks, taking advantage of the chelating ability of pH-responsive carboxylic groups in PAA. Increasing the pH ionizes carboxyls, increases the porosity in pHEMA/PAA, and leads to the co-presence of enol and enolate forms of vinylpyrrolidone (VP), changing the macroporosity and decreasing the mesh size in pHEMA/PVP. The ion–matrix interaction is stronger in pHEMA/PAA, where the process occurs through an initial diffusion-limited step followed by diffusion in smaller pores or adsorption by less available sites. In pHEMA/PVP, the uptake is probably controlled by adsorption as expected, considering the porogen role of PVP in the network. Upon application of the SIPNs loaded with tetraethylenpentamine (TEPA) onto corroded bronze, copper oxychlorides dissolve and migrate inside the gels, where Cu(II) ions form ternary complexes with TEPA and carboxylates in PAA or carbonyls in PVP. The removal of oxychlorides is more effective and faster for pHEMA/PAA than its /PVP counterpart. The selective action of the gels preserved the cuprite layers that are needed to passivate bronze against corrosion, and the pH-responsive behavior of pHEMA/PAA allows full control of the uptake and release of the Cu(II)–TEPA complex, making these systems appealing in several fields even beyond Cultural Heritage conservation (e.g., drug delivery, wastewater treatment, agricultural industry, and food chemistry).

## Introduction

The conservation of Cultural Heritage has deep societal and economic implications because well-preserved and accessible works of art constitute both a drive for social inclusion and an important resource to promote tourism and job creation.^1^ Metallic objects and artifacts constitute a vast part of the artistic and architectural production spanning over millennia in the history of mankind. They are typically affected by several degradation processes that can significantly alter their appearance and integrity. In particular, copper-based artifacts are affected by corrosion phenomena that induce the formation of a complex patina on their surface, usually characterized by the presence of copper oxychlorides (i.e., atacamite and its polymorphs) responsible for the so-called “bronze disease”, a cyclic degradative process able to consume the objects up to their complete disgregation.^2^ The removal of corrosion products is thus a fundamental operation in conservation practice, but still an open challenge that needs feasible solutions. Traditionally, cleaning is performed by mechanical (vibrating or abrasive tools, ultrahigh-pressure water), optical (laser ablation), and chemical wet methods (bases, acids, and complexing agents).^3^ However, these approaches involve several risks for the artifacts, unless time-consuming protocols are adopted: mechanical treatments and cleaning fluids in their bulk state are invasive and scarcely selective, while laser ablation can trigger heating processes on the surface of artifacts, generating defects in the metal.^4^ The confinement of cleaning fluids is an optimal strategy to achieve controlled removal without risks for the objects,^5^ but traditional thickeners used in restoration (e.g., cellulose derivatives, viscous dispersions of poly(acrylic acid)) are either not sufficiently retentive or exhibit poor mechanical properties and tend to leave residues on the treated surfaces.^6^ In the last decade, chemical hydrogels have been proposed as optimal matrices to confine fluids for the safe cleaning of works of art.^7−11^ In particular, chemical semi-interpenetrated polymer networks (SIPNs) of poly(2-hydroxyethyl methacrylate) (pHEMA) and different linear polymers show tunable physicochemical and mechanical properties depending on the type of interpenetrating polymer chains. Combining pHEMA and poly(vinylpyrrolidone) (PVP), for instance, results in networks that exhibit the ideal mechanical properties of pHEMA and the hydrophilicity and water loading capability of PVP. Thanks to these characteristics, the pHEMA/PVP gels have been successfully applied on numerous case studies to remove soil, aged varnishes/adhesives, or overpainting (e.g., vandalism) from the surface of sensitive works of art;^9,10^ the gels can be applied repeatedly with no risks to delicate substrates and simply removed in one piece without leaving detectable residues. These features could be particularly advantageous on corroded surfaces that are usually brittle and mechanically weak, where the removal or peeling of surface layers (e.g., using film-forming polymeric dispersions^12^) might be risky for the artifact integrity. However, a fundamental requirement in the treatment of degraded bronze artifacts is that copper ions in the corrosion layers must be captured and effectively retained in a sorbent matrix. While the pHEMA/PVP might show some effectiveness, thanks to the intrinsic ability of VP moiety to form complexes with metal ions,^13^ we propose here, alternatively, a novel SIPN, where pHEMA is interpenetrated with poly(acrylic acid) (PAA), whose ability to give strong coordination bonds with metals *via* carboxylate groups at pH higher than 7^14^ is expected to boost the capture and retention of copper. To the best of our knowledge, interpenetrated networks of pHEMA and PAA are still vastly unexplored,^15^ and this is the first time a pHEMA/PAA SIPN, rather than classical copolymers,^16^ is formulated and physicochemically characterized.

Because Cu(II) ions must be removed from the solid lattice of corrosion products (e.g., oxychlorides, oxides, and carbonates) before they can be fixed in the gel matrix, we loaded the SIPNs with tetraethylenpentamine (TEPA), a highly selective chelating agent whose Cu(II) complex has a stability constant (log *K*_f_ = 22.8 at 25 °C^17^) 4 orders of magnitude higher than that formed by the tetrasodium salt of ethylenediaminetetraacetic acid (EDTA, Y^4–^) (log *K*_f_ = 18.8 at 25 °C and 1 M^18^), which is traditionally employed by conservators in the removal of copper corrosion products. We investigated the effects of pH variation in the SIPNs, and their mutual interaction with copper ions and TEPA, by swelling the gels either in water (at pH 6, 8, and 12) or in a TEPA aqueous solution (pH 12) and analyzing them through small-angle X-ray scattering (SAXS), two-dimensional (2D) Fourier transform infrared (FTIR) spectroscopy imaging, and scanning electron microscopy (SEM), so as to monitor chemical and structural differences at the micro- and nanoscales. Differential scanning calorimetry (DSC) and thermogravimetric analysis (TGA) were employed to evaluate the solvent content of the gels and the properties of water entrapped in the polymeric networks. The Cu(II) ion adsorption kinetics of pHEMA/PVP and pHEMA/PAA SIPNs at different pH values were investigated and compared, highlighting the effect of the structure and functional groups of the gels on the adsorption process. Finally, the two classes of gels were applied on a corroded bronze mock-up, and their ability to remove copper corrosion patinas was critically compared.

## Experimental Section

### Materials

2-Hydroxyethyl methacrylate (HEMA) (purity 99%), poly(acrylic acid) (PAA) (average Mn ≈ 1200 kDa), azoisobutyronitrile (AIBN) (purity 98%), *N*,*N*-methylene-bis(acrylamide) (MBA) (purity 99%), poly(vinylpyrrolidone) (PVP) (average Mn ≈ 1300 kDa), tetraethylenepentamine (TEPA) (purity ≥ 95%), sodium hydroxide pellets (purity 97%), and copper(II) chloride dihydrate (purity > 99.0%) were purchased from Sigma-Aldrich and used as received. Potassium dihydrogen phosphate (purity ≥ 99.0%) and dipotassium hydrogen phosphate (purity ≥ 98%) were purchased from Merck and used as received to prepare a pH 8 buffer. Water was purified by a Millipore Milli-Q gradient system (resistivity > 18 MΩ·cm).

### Synthesis of Hydrogels

The pHEMA/PVP SIPN was prepared by radical polymerization as reported by Domingues et al.^11^ Some variations in the synthetic process were adopted: the HEMA/PVP ratio was changed from 30/70 to 27.5/72.5 (% w/w), the water content in the pre-gel solution was 62.2% instead of 65%, while the cross-linker concentration was halved. These changes, all together, were adopted so to produce slightly softer and more flexible gel sheets able to easily adapt onto the rough surface of corrosion patinas.

The pHEMA/PAA SIPN was synthesized by adding an aqueous solution of PAA to HEMA monomer and AIBN. The ratio between the mass of pHEMA and PAA (96.5/3.5% w/w) was chosen to have a molar ratio between −OH and −COOH groups of 16/1, which proved to be an optimal condition to favor the synthetic process, yielding gels with good mechanical properties. After sonication and degassing, the mixture was transferred between two glassy covers and polymerized at 60 °C for 4 h. After the reaction, a 2 mm thick flat hydrogel sheet was obtained; the gel was then washed by renewing water once a day for 7 days to remove residues of unreacted monomers and free PAA molecules. Table 1 shows the composition of the two hydrogels.

**Table 1:** Composition of pHEMA/PAA and pHEMA/PVP SIPNs

SIPN	pHEMA/PAA	SIPN	pHEMA/PVP
HEMA (wt %)	52.5	HEMA (wt %)	10.3
water (wt %)	45.2	water (wt %)	62.2
MBA (wt %)		MBA (wt %)	0.4
AIBN (wt %)	0.4	AIBN (wt %)	0.1
PAA (wt %)	1.8	PVP (wt %)	27.0
HEMA/PAA ratio (%w/w)	96.5/3.5	HEMA/PVP ratio (%w/w)	27.5/72.5

The SIPNs were swollen in water, reaching a stable pH of 6.3. Small pieces (5 × 5 × 0.2 cm^3^) were cut and swollen with water at pH 8 and 12 (adjusted with a sodium hydroxide 1 N solution), and in a water solution of TEPA (20% w/w, pH = 12). In all cases, the gels were placed in the NaOH or TEPA solutions for at least 5 days, using an excess of solution compared to the gel’s mass, to make sure that the SIPNs equilibrated completely.

### Thermal Analyses

TGA was carried out with an SDT Q600 (TA Instruments) and a balance sensitivity of 0.1 μg. Measurements were performed in a nitrogen atmosphere with a flow rate of 100 mL/min. The samples were put in open aluminum pans, and the analyses were performed with a heating rate of 10 °C/min from 25 to 450 °C.^19^

The equilibrium water content (EWC) and the equilibrium solvent content (ESC) were calculated as follows (eq 1)
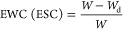
1where *W* is the weight of the equilibrated sample and *W*_d_ is the weight of the dry sample. The values of *W*_d_ were experimentally determined from TGA analysis, considering the weight of the sample at ca. 200 and 300 °C to quantify the EWC (SIPNs swollen in water) and the ESC (SIPNs swollen in TEPA), respectively.

DSC was performed with a Q2000 Calorimeter (TA Instruments). The temperature range was from −80 to 200 °C with a scan rate of 2 °C/min; sealed stainless-steel pans were used. From the DSC curves, it is possible to determine the different types of water present in the hydrogels.^20^ Water in porous systems like gels can be classified as nonfreezing bound water and free (i.e., bulklike) water.^21^ The nonfreezing water forms hydrogen bonds with the functional groups of the polymer, rather than with other water molecules (as would be necessary for water to freeze); bulk water has the same properties of pure water and can bind with other water molecules to form ice crystals when the temperature is around 0 °C. It is possible to determine the free water index (FWI) according to the following equation (eq 2)
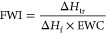
2where Δ*H*_tr_ (J/g) is the heat of transition obtained by the integral of melting peaks around 0 °C in the DSC curves and Δ*H*_f_ is the theoretical value of the specific enthalpy of fusion of water at 0 °C (333.6 J/g^22^).

### Scanning Electron Microscopy

SEM investigation was performed on the sponges, obtained by freeze-drying thin slices of the hydrogels. An FEG-SEM ∑IGMA (Carl Zeiss, Germany) was used to acquire the images using an acceleration potential of 2 kV and a working distance of 3 mm. Before carrying out the analysis, the samples were coated with a thin layer of gold using an Auto Sputter Coater (Agar Scientific).

### 2D Image Analysis

SEM images were analyzed, first, through the chord length distribution approach, to obtain the average dimension of the pores and walls of the gels. More specifically, the MATLAB algorithm developed by MacIver^23^ was readapted to the present investigation and used as detailed. Each chosen SEM micrograph was converted in a gray-scale image, then contrast-enhanced, and finally binarized; the resulting black and white (b/w) image is simplified with respect to the original micrograph. At this point, the MATLAB algorithm drew a set of 10 000 randomly oriented lines on the 2D, b/w image. Segments, called chords, form when lines cross phase boundaries (i.e., when lines pass from black to white areas, or *vice versa*). The frequency of chords of a certain length, *f*(*R*), is plotted against their dimension, *R* (μm). The minimum detectable chord length was set to 2 pixels (0.18 μm) for gels with smaller pores (pHEMA/PAA at pH 8 and 12, pHEMA/PVP at pH 12) and to 5 pixels (0.3 μm) for pHEMA/PVP at pH 6 and pHEMA/PVP at pH 8, to reduce noise. The data trend was independent of the minimum chord value.

Final datasets were smoothed through the Igor Pro built-in function to improve readability. For the pores and walls of the gels, the decay in the frequency of the most abundant chords evolves according to exponential functions^24^
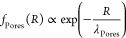
3
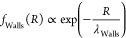
4

In both equations, 1/λ represents the slope of the function in a semilog graph. λ is called persistence length and represents a characteristic length scale of the gel domains. Frequencies as low as 0.001 were considered for the evaluation of λ. For the analysis of chord lengths, frontal SEM pictures were considered, so as to avoid artifacts in the length distributions.

The obtained characteristic dimensions were compared to the average pore diameter obtained, on the same images, through ImageJ software^25^ (“Count Particles” analysis with a minimum diameter of ca. 0.01 μm, i.e., comparable to the minimum chord dimension considered in the chord analysis), and to *R* values at a cumulative frequency of 50% over the total population. From this comparison, uncertainties on λ were estimated from the fitting or taken as the 10% at least of the values of λ. To improve the statistics, ImageJ analysis was performed on at least three SEM images at different magnifications. Results are listed in the Supporting Information (SI).

### Small-Angle X-ray Scattering

Small-angle X-ray scattering analysis (SAXS) was carried out with a HECUS S3-MICRO SWAXS camera, equipped with a System3 Kratky camera (Hecus, Graz) and two position-sensitive detectors (PSD-50M) containing 1024 channels with a width of 54 μm. Microfocus source (50 W, Cu anode, Oxford Instruments) emits radiation with the wavelength of the *K*_α_-line given by λ = 1.542 Å. The *K*_β_-line is removed by FOX-3D single-bounce multilayer point focusing optics (Xenocs, Grenoble). The sample-to-detector distance was 281 mm. The volume between sample and detector was kept under vacuum to minimize the scattering from air. The camera was calibrated in the small-angle region using silver behenate, which is known to have a well-defined lamellar structure (*d* = 58.38 Å).^26^ Scattering curves were acquired in the *Q*-range between 0.01 and 0.55 Å^–1^, where *Q* = (4π sin θ)/λ, and 2θ is the scattering angle. Samples were placed into demountable cells, with Kapton film used as windows. The temperature control was set to 25 °C by a Peltier element, with an accuracy of ±0.1 °C. All the scattering curves were corrected for the empty cell and water contribution considering relative transmission factors.

### 2D FTIR Imaging

2D FTIR imaging analysis was carried out on gel sponges (obtained after freeze-drying from the corresponding hydrogels) and bronze mock-ups, using a Cary 620–670 FTIR microscope, equipped with an FPA 128 × 128 detector (Agilent Technologies). This setup allows the highest spatial resolution currently available to FTIR microscopes. The spectra were recorded directly on the surface of the samples (gels, corroded bronze coins, or the Au background) in reflectance mode, with an open aperture and a spectral resolution of 4 cm^–1^, acquiring 128 scans for each spectrum. A “single-tile” analysis results in a map of 700 × 700 μm^2^ (128 × 128 pixels), and the spatial resolution of each imaging map is 5.5 μm (i.e., each pixel has dimensions of 5.5 × 5.5 μm^2^). Multiple tiles can be acquired to form mosaics. To improve the readability of the spectra, the background noise was reduced using the “smooth” tool (set at 11) of the Igor Pro software (Wavemetrics), taking care not to alter any diagnostic information deemed useful to this investigation. In each 2D map, the intensity of characteristic bands of the gels, or of bronze corrosion products, was imaged. The chromatic scale of the maps shows increasing absorbance of the bands as follows: blue < green < yellow < red.

### Cu(II) Adsorption Kinetics of the SIPNs

Cu(II) adsorption kinetics were carried out on 500 mL of copper chloride solutions (10^–4^ M) at two different pH values (6 and 8); the solution at pH 8 was obtained using a phosphate buffer solution. The Cu(II) adsorption at pH 12 was not evaluated owing to the precipitation of copper hydroxide. Pieces of pHEMA/PAA and pHEMA/PVP SIPNs were cut (5.0 × 5.0 × 0.2 cm^3^), blotted with paper to remove any excess surface water, and weighed. Kinetic measurements started when the gel was immersed in the copper chloride solution and stopped after 180 min; this time interval was chosen as it widely covers real application times (generally no more than 2–3 h); 1 mL aliquots were taken from the solution at set times and analyzed with a PerkinElmer Model AAnalyst 100 Flame Atomic Absorption Spectrometer (F-AAS) equipped with a 10 cm air–acetylene burner. The instrument was equipped with a multielement hollow cathode lamp and a deuterium lamp for background correction.

The instrument was operated under the conditions recommended by the manufacturer: lamp current of 30 mA, wavelength of 324.8 nm, slit width of 0.2 nm. The standard solutions and samples were introduced into the flame atomic absorption spectrophotometer by means of a standard nebulizer and flow spoilers. The absorbance of the samples was measured in triplicate against the blank solution, and the average of the three measurements was used as the analytical signal. Standard solutions, for Cu^2+^ calibration, were daily prepared in polyethylene vials by diluting a Cu^2+^ stock standard solution (1000 mg L^–1^) purchased from Merck (Darmstadt, Germany) with ultrahigh-purity water (UHQ) of resistivity > 18 MΩ·cm (Milli-Q system by Millipore, Billerica, MA).

### Bronze Mock-Ups and Cleaning Procedure

To evaluate the effectiveness of the gels, cleaning tests were carried out on an artificially aged bronze coin, which was provided by CNR-ISMN (Rome, Italy). The artificial aging procedure, developed by Ingo et al., produces corrosion patinas that are similar in appearance and composition to those of archeological bronze artifacts.^27^ Small pieces of the two gels (1 × 1 × 0.2 cm^3^) were loaded with an aqueous TEPA solution (20% w/w) at pH 12. The gels were applied on the coin surface up to 60 min, covered with parafilm to limit evaporation of fluid from the polymer network. During the application, the strong blue color of the gels indicates the absorption of Cu(II) ions and the formation of Cu(II) complexes. After the treatment, the coin substrate was rinsed with water and air-dried. 2D FTIR imaging was carried out on the coin surface before and after the application of the gels, checking the presence of corrosion products and gel residues.

## Results and Discussion

**Figure 1:**
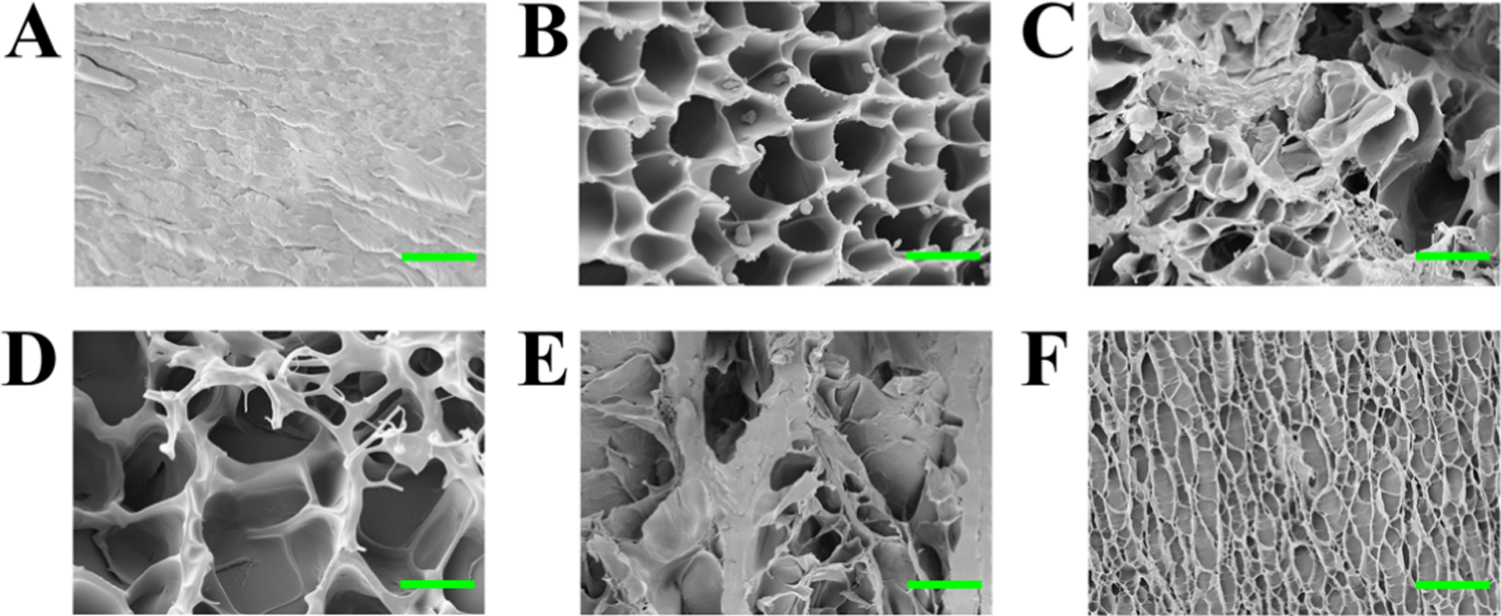
SEM images of pHEMA/PAA (A–C) and pHEMA/PVP (D–F) sponges obtained after freeze-drying from the corresponding hydrogels swollen in water at pH 6 (A, D), 8 (B, E), and 12 (C, F). Scale bar is 10 μm.

SEM images show the influence of pH on the architecture of different gels (Figure 1). At pH 6, pHEMA/PAA SIPN has a compact structure and does not exhibit any porosity at the microscale (Figure 1A), while increasing the pH to 8, a quite homogeneous porosity develops in the 7–10 μm range (Figure 1B). A more heterogeneous structure is noted at pH = 12, where pores have an irregular shape and a broader size distribution (Figure 1C). The pHEMA/PVP SIPNs exhibit a well-developed porosity in the investigated pH range (Figure 1D–F); interestingly, increasing the pH up to 12 results in elongated pores arranged in a quite ordered pattern with a preferential direction (Figure 1F).

**Figure 2:**
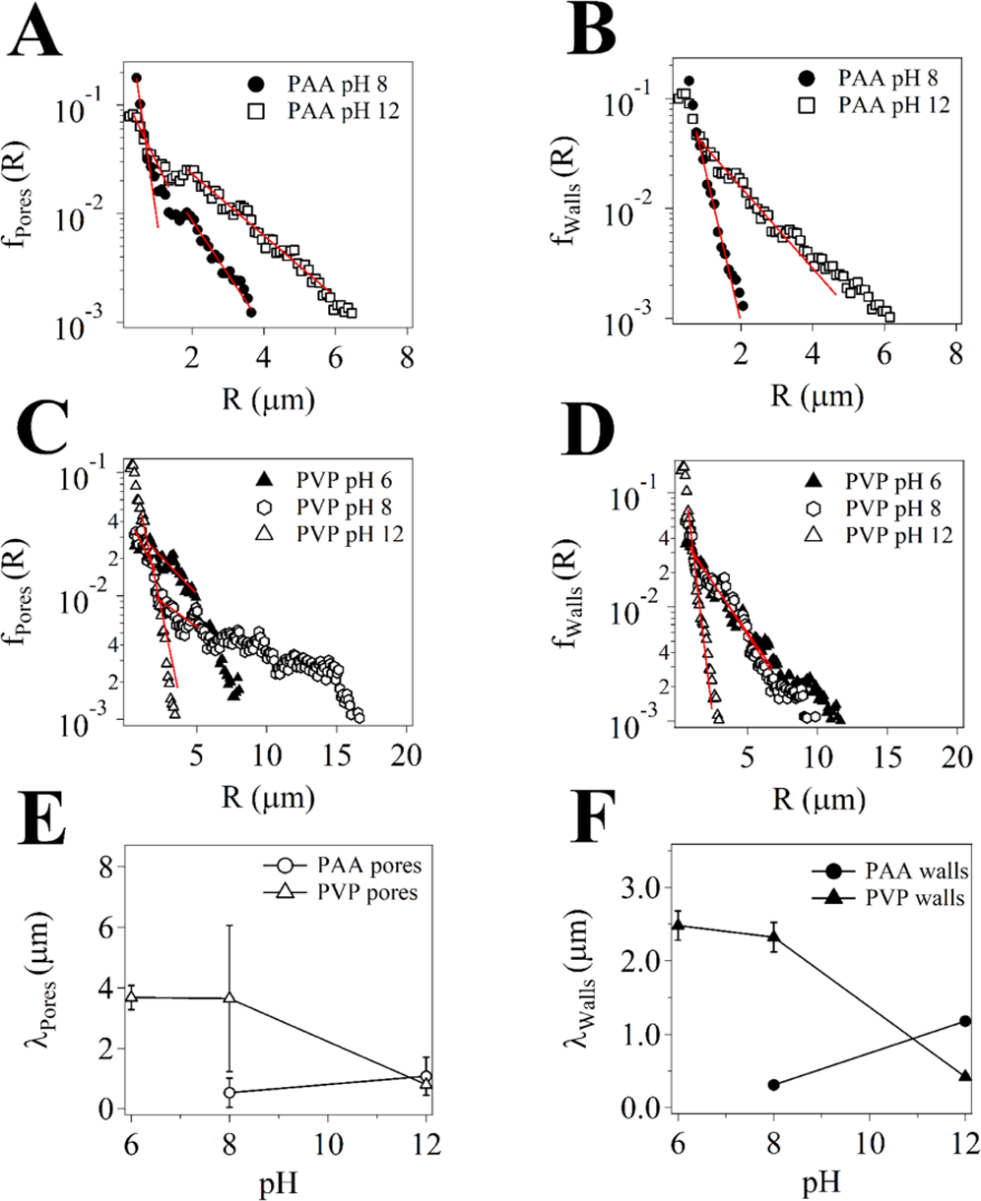
Chord length distributions, *f*(*R*), of the pHEMA/PAA and pHEMA/PVP sponges obtained after freeze-drying from the corresponding hydrogels (A–D) and plots of relative persistence lengths λ (calculated from the slopes of curves; see Tables S1 and S2) *vs* pH. (A, B) pHEMA/PAA gels at pH 8 and 12; (C, D) pHEMA/PVP gels at pH 6 and 8; (E, F) trends of λ (for pores and walls) *vs* pH; λ_Pores_ of pHEMA/PAA gels at pH 6 and 8, and pHEMA/PVP gels at pH 8 is an average of the two values obtained for smaller and larger pores; their error bars show the distance between the two values. Error bars account for uncertainties of 10% at least; when not shown, they are included in the markers.

Quantitative information about the average pore and wall dimensions were obtained by implementing the chord length distribution analysis. This method allows extracting the characteristic length scales, λ, from a micrograph of a biphasic media. Such information is obtained by drawing a set of randomly oriented lines on binarized, frontal SEM micrographs (Figure S1). Chords are then defined from the intersection of each line with the phase boundary. The frequency of occurrence of chords with a certain length, *f*(*R*), plotted against the chord dimension, *R*, gives information on the sample morphology and, in particular, returns the characteristic dimensions present in the hydrogel in terms of pores and walls. The distributions for pHEMA/PAA and pHEMA/PVP gels, equilibrated at pH 6, 8, and 12, are shown in Figure 2A–D, respectively. The pHEMA/PAA gel at pH 6 was not included, being nonporous at the investigated length scales (see Figure 1a). General information about the maximum pore size of each sample can be obtained by considering the intersection of the distributions with the x-axis (Figure 2): in pHEMA/PAA gels, chords describing pores extend up to ∼3.5 μm at pH 8, and to ∼6.5 μm at pH 12; pHEMA/PVP pores, on the other hand, are described by chords whose dimension varies largely with pH: at pH 6, the maximum pore size is ∼7.5 μm, increasing to ∼16.5 μm at pH 8 and shrinking to ∼3.5 μm when pH reaches 12 units. In pHEMA/PAA gels, the largest wall thickness is ∼2 μm at pH 8 and ∼6 μm at pH 12. In pHEMA/PVP, chords describing walls are almost superimposed at pH 6 and 8, with their maximum being around 10 μm at pH 8 and 11.5 μm at pH 6. pHEMA/PVP at pH 12 has thinner walls, their maximum dimension being around 3 μm. Minimum dimensions accessible in all cases are limited by the resolution of the SEM experiment (i.e., pixel size).

For all of the investigated samples, *f*(*R*) of both pores and walls displays a slight initial increase at the first two to three points of the curves, followed by one or more exponential decays (Figure 2A–D). Each portion of the curves that showed a clearly observable trend was fitted according to an exponential decay (see eqs 3 and 4). The variation with pH of the persistence length of pores and walls (λ_Pores_ and λ_Walls_, obtained from the fittings) is shown in Figure 2E,F for all of the investigated samples and corresponding values listed in Tables S1 and S2 (see the SI). For systems characterized by two characteristic slopes, an average value was considered, with its standard deviation.

Regarding pore distributions, the pHEMA/PAA gels clearly show two different slopes, describing the trend of smaller and larger pores (Figure 2A,E). pH affects pore dimensions, which are larger at pH 12: the deprotonation of carboxyl groups in PAA leads to an increased electrostatic repulsion, enhancing the swelling of the structure of the gels. The pHEMA/PVP gels have, in general, larger pores than pHEMA/PAA networks, except at pH 12 (see Figure 2C,E). For these systems, two slopes were considered only for the sample at pH 8. In general, the average λ does not vary significantly passing from pH 6 to 8 (Table S1), even though the pore size distribution is larger at pH 8. The λ_Pores_ value decreases, instead, at pH 12. The latter behavior could be explained considering that, at highly alkaline pH values (≫10), the enol tautomer of PVP is predominant and can lose a proton to form an enolate;^28^ the enol and enolate forms are likely to interact tightly, leading to a more shrunk pore network.

Regarding wall size distributions, λ_Walls_ is larger for pHEMA/PVP gels than pHEMA/PAA, again except for the gels at pH 12 (Figure 2F). Noticeably, the chord distributions of both pores and walls for pHEMA/PVP gel at pH 12 are characterized by a steep slope: this indicates that pores are elongated, and walls are thin and threadlike, in agreement with the morphology of the porous networks seen in the SEM images.

To test the soundness of the λ values obtained, the average diameters (*D*) of pores on the same images were calculated with the ImageJ software (Table S1); moreover, the values of *R* at 50% of cumulative frequency, for both pores and walls, was considered to improve the statistics (Figure S2 and Tables S1 and S2). In general, the average *D* and *R* (50%) are slightly lower than λ, as smaller pores affect them more. Nevertheless, the characteristic dimensions obtained with the three methods are in agreement. Pore dimensions on SEM images at different magnifications were also calculated by ImageJ. The average diameters, listed in Table S1, are consistent with the previous results.

**Figure 3:**
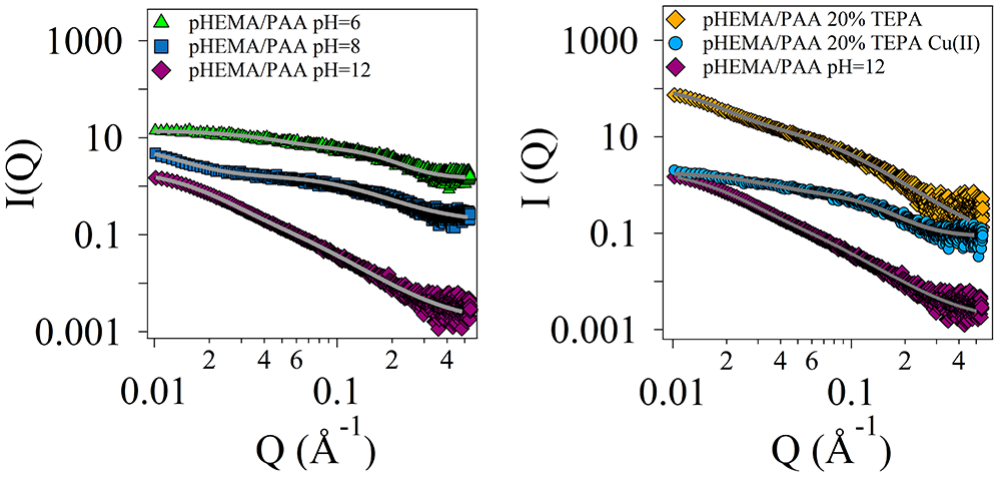
SAXS curves and fitting (gray lines) of pHEMA/PAA SIPNs swollen in water (left) at pH 6 (green triangles), 8 (dark blue squares), and 12 (purple diamonds), and loaded with TEPA (right, orange diamonds) or TEPA and copper II ions (right, blue circles). For the sake of clarity, data are shifted along the *y* axis.

**Figure 4:**
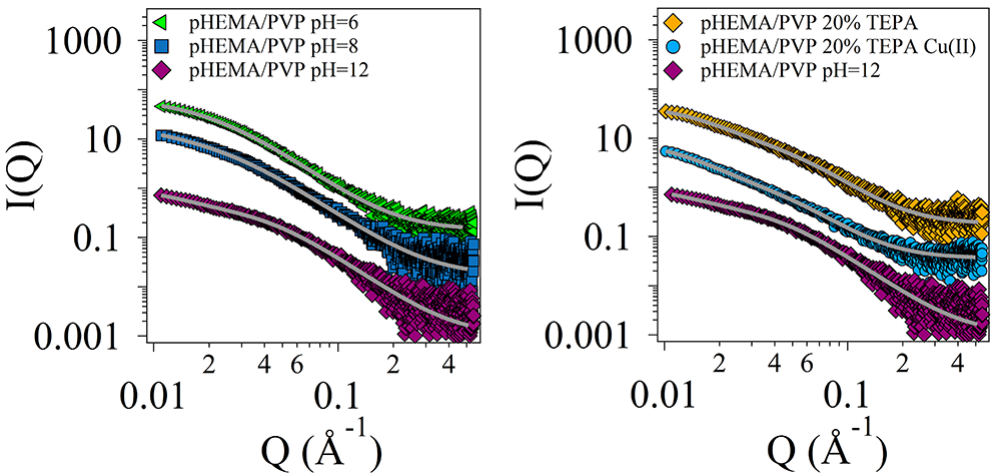
SAXS curves and fitting (gray lines) of pHEMA/PVP SIPNs swollen in water (left) at pH 6 (green triangles), 8 (dark blue squares), and 12 (purple diamonds) and loaded with TEPA (right, orange diamonds) or TEPA and copper II ions (right, blue circles). For the sake of clarity, data are shifted along the *y* axis.

While chord analysis provided quantitative details about the micron-sized porosity of the gels, SAXS experiments were carried out to investigate modifications in the SIPNs at the nanoscale. In this case, changes could be induced by pH variations, the presence of TEPA, and the co-presence of TEPA and Cu(II) ions (following the application of the gels onto corroded bronze coins). Figures 3 and 4 show the SAXS curves of pHEMA/PAA and pHEMA/PVP hydrogels at pH 6, 8, and 12, and loaded with TEPA or with TEPA and Cu(II) ions. All of the SAXS curves were modeled using a generalized version of the Debye–Bueche approach,^29^ with two *Q*-dependent contributions and an instrumental flat background^30^ (eq 5)
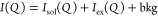
5

The first contribution *I*_sol_(*Q*) is a generalized version of the Ornstein–Zernike equation (eq 6)
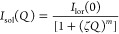
6where *I*_lor_(0) is the scattering intensity at *Q* = 0, dependent on the contrast between the polymer and the solvent and on the volume fraction of the polymer in the gel; ζ is the characteristic average mesh size (or correlation length) of the network; and *m* is the Porod exponent associated with the solvation term. The second contribution *I*_ex_(*Q*) is related to the excess of scattering at a low q caused by the solid-like inhomogeneities of the polymeric network (eq 7)
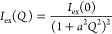
7where *I*_ex_(0) represents the excess intensity at *Q* = 0 and *a* is the length scale that characterizes gel inhomogeneities.

The fitting parameters of the SAXS curves of pHEMA/PAA SIPNs swollen in water are reported in Table 2. The average mesh size increases moving from pH 6 to 12, confirming that the electrostatic repulsion between PAA chains when the carboxyl groups are ionized leads to the stretching of the polymer chains.^31^ Consistently, an increase of EWC is observed with increasing pH values, in agreement with other publications where the mesh dimension is strongly related to the equilibrium water content.^32^ The Porod exponent has a value of ca. 3.8 for the hydrogel at pH 6, suggesting that the polymer network is collapsed, while at pH 8 and 12, the exponent decreases to 2.3 for both systems, as a result of the increased interaction between the polymer chains and the solvent (i.e., theta solvent conditions are approaching where *m* = 2.0). The dimension of inhomogeneities increases from 1.7 nm at pH 6 to 7.3 nm at pH 8, and ca. 5 at pH 12, suggesting that the increase in water content (higher EWC) leads to a less homogeneous SIPN at the nanoscale.^11^

**Table 2:** Fitting Parameters Obtained from SAXS Curves of pHEMA/PAA SIPNs

	pH = 6	pH = 8	pH = 12	20%TEPA	20%TEPA Cu(II)
*I*_lor_(0)	1.37 ± 0.05	2.77 ± 0.05	11.2 ± 0.6	9.5 ± 0.2	3.36 ± 0.09
ζ (nm)	0.6 ± 0.1	0.8 ± 0.1	1.8 ± 0.3	1.1 ± 0.2	0.8 ± 0.1
*m*	3.7 ± 0.1	2.3 ± 0.1	2.3 ± 0.1	2.4 ± 0.1	3.3 ± 0.1
*I*_ex_(0)	2.7 ± 0.1	13 ± 1	144 ± 1	79.3 ± 0.9	7.9 ± 0.2
*a* (nm)	1.7 ± 0.1	7.3 ± 0.2	5.0 ± 0.2	5.5 ± 0.2	2.5 ± 0.1
bkg	0.44 ± 0.01	0.34 ± 0.01	0.11 ± 0.01	0.03 ± 0.01	0.54 ± 0.01

When pHEMA/PAA is loaded with an aqueous solution of TEPA (20%, pH = 12), the mesh size and the dimension of solid-like inhomogeneities are smaller than those of gels simply loaded with water at the same pH, while the Porod exponent is slightly higher. These changes are ascribable to interactions between TEPA and the carboxylic groups in PAA, where molecules of TEPA might interpose between chains of PAA, screening the repulsion between carboxylate groups and making the SIPN tighter. A further decrease of the mesh and inhomogeneities size is observed in the presence of Cu(II) ions. Cheng et al. reported a decrease of the radius of gyration (*R*_g_) for a poly(*N*-isopropylacrylamide) copolymer hydrogel that adsorbed Cu(II), and such a change was ascribed to the formation of complexes between the ions and chelating groups in the polymer chains.^33^ In our case, the lower mesh is likely due to the formation of complexes between Cu(II) and ionized carboxylic groups of PAA, e.g., each copper ion coordinating with two −COO^–^ groups from different chains.^34^ Besides, a small decrease in the EWC (about 6%) is observed, in agreement with the lower mesh size value.^32^ Finally, the increase in the Porod exponent (see Table 2) indicates a transition to a denser aggregate structure.

Regarding pHEMA/PVP SIPNs (see Table 3 and Figure 4), the average mesh size at pH = 6 is in agreement with previous studies^11^ and remains unchanged at pH = 8, while at pH = 12, it decreases by about 1 nm, in agreement with the decrease of the persistence length of pores and walls highlighted by the chord analysis.

**Table 3:** Fitting Parameters Obtained from SAXS Curves of pHEMA/PVP SIPNs

	pH = 6	pH = 8	pH = 12	20%TEPA	20%TEPA Cu(II)
*I*_lor_(0)	25.3 ± 11.3	37.7 ± 5.4	15.5 ± 0.6	7.3 ± 0.4	10.8 ± 0.5
ζ (nm)	3.4 ± 0.5	3.8 ± 0.2	2.3 ± 0.1	1.9 ± 0.1	2.3 ± 0.1
*m*	2.4 ± 0.1	2.4 ± 0.1	2.6 ± 0.1	2.7 ± 0.1	2.6 ± 0.1
*I*_ex_(0)	68 ± 9	62 ± 3	35 ± 2	46 ± 1	89 ± 1
*a* (nm)	4.9 ± 0.6	6.9 ± 0.8	6.5 ± 0.4	5.1 ± 0.1	6.2 ± 0.1
bkg	0.20 ± 0.01	0.09 ± 0.01	0.04 ± 0.01	0.20 ± 0.01	0.38 ± 0.01

As stated above, the formation of enolates in PVP, induced by the high pH, might lead to an enhancement of inter- and intramolecular hydrogen bonds with the residual enol groups, resulting in a smaller mesh size and in a more compact structure, as also suggested by the slight increase of the Porod exponent. The gel swollen in a water solution of TEPA (pH = 12) exhibits the smallest value of ζ (ca. 2 nm) and the highest value of *m* (2.7) (see Table 3). When Cu(II) is absorbed in the hydrogel, similarly low values are found. This behavior can be explained considering that enolate groups are able to interact with TEPA molecules and Cu(II) ions, closing together in the formation of complex structures.

The study of the amount and types of water loaded in the hydrogels provided information on the absorption and permeation properties of these systems (see SI, Figures S3–S6 and Tables S3–S4). The EWC and FWI in pHEMA/PAA SIPNs increase with pH (see Table S3), owing to the ionization of the carboxyl groups to carboxylates (which are more hydrophilic) and consequent pores enlargement. In the case of pHEMA/PVP SIPNs, both the EWC and FWI remain unchanged at different pH (Table S4), and these systems have higher values than pHEMA/PAA, which is explained considering the high relative content of PVP, a highly hydrophilic polymer.

It must be noticed that for both systems, there is a significant decrease in the heat of the melting transition (Δ*H*_tr_; see Tables S3 and S4) when the gels are uploaded with the TEPA solution and when Cu(II) ions are absorbed in the gels. The FWI decreases accordingly. In the case of TEPA-loaded gels, this was ascribed to strong hydrogen bonding taking place between the amine and water molecules, which also explains the lower critical solution temperature (as previously observed in amine-water solutions).^35^ When the gels absorb the copper ions, the further decrease was explained considering that part of the bulk water molecules coordinates with the metal ions, participating in the formation of complexes.

**Figure 5:**
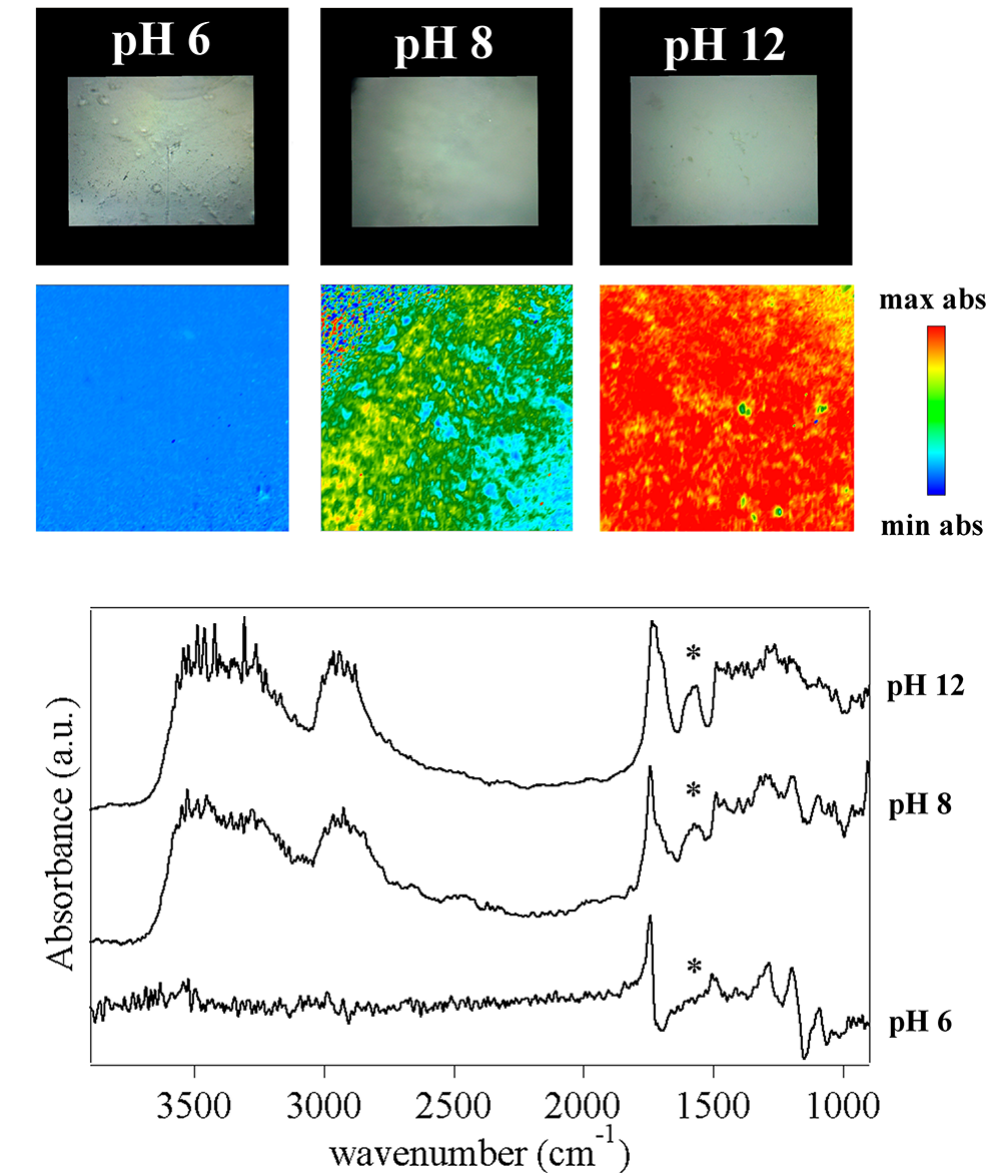
FTIR 2D imaging of pHEMA/PAA sponges obtained from hydrogels that were swollen with water at different pH values. Below each visible image, the corresponding 2D FTIR Imaging map shows the intensity of the band at 1578 cm^–1^ (−COO^–^ antisymmetric stretching of carboxylate groups in PAA). All maps have dimensions of 700 × 700 μm^2^. The bottom panel shows representative spectra of pixels (5.5 × 5.5 μm^2^) in the corresponding 2D imaging map. The symbol highlights the antisymmetric stretching of the −COO^–^ groups in PAA, whose intensity increases passing from pH 8 to pH 12.

Further information on the chemical changes of the SIPNs at different pH, and upon loading of TEPA and Cu(II) ions, was provided by FTIR 2D imaging. At pH 6, the spectra of the pHEMA/PAA SIPN exhibit features typical of reflective surfaces, with derivative-shaped peaks and distorsions (see Figure 5). In particular, the OH stretching and CH stretching bands of pHEMA and PAA are not observable, and the main peaks are those of C=O stretching (derivative shape, maximum at 1745 cm^–1^), CH_2_ bending (1496 cm^–1^), C–O stretching (1294 cm^–1^), and C–O–C stretching (derivative shape, maximum at 1197 cm^–1^ and 1092 cm^–1^).^36,37^ At alkaline pH values, the spectra change significantly and their aspect resembles much more closely that of standard transmission spectra. This indicates that a change in the refractive index of the gel has occurred, following the neutralization of the carboxyl groups in PAA and the rearrangement of the polymer chains. Both the OH and CH stretching bands are clearly observable, and the C=O stretching peak shows two components around 1740 (pHEMA) and 1715 (PAA) cm^–1^.^36,37^ The spectra show a new peak at 1578 cm^–1^, assigned to the antisymmetric stretching of the −COO^–^ groups in PAA, whose intensity increases passing from pH 8 to pH 12 (as shown in the FTIR maps in Figure 5). Besides, while at pH 8, the carboxylate groups concentrate in domains ranging from tens to hundreds of microns, at pH 12, they are homogeneously distributed across the gel matrix.

**Figure 6:**
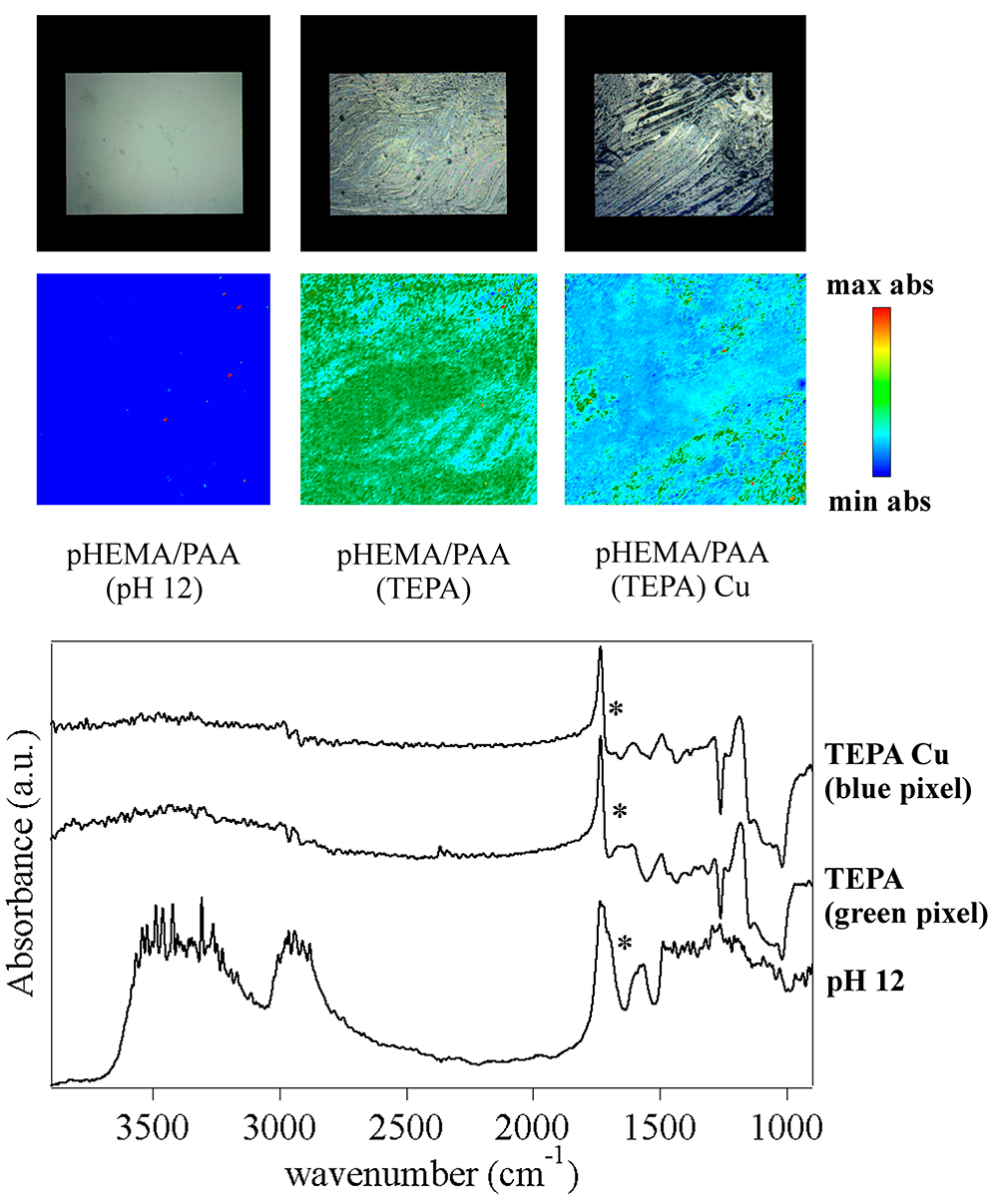
(Top) FTIR 2D imaging of sponges obtained from pHEMA/PAA hydrogels: (top left) swollen with water at pH 12; (top center) sponges obtained from gels swollen with a TEPA solution (20%); (top right) sponges obtained from gels that were swollen with the TEPA solution and then placed on a bronze coin mock-up containing Cu(II) corrosion products. Below each visible image, the corresponding 2D FTIR Imaging map shows the intensity of the band at 1660 cm^–1^ (assigned to the −COO^–^ antisymmetric stretching of carboxylate groups in PAA). All maps have dimensions of 700 × 700 μm^2^. The bottom panel shows representative spectra of pixels (5.5 × 5.5 μm^2^) in the corresponding 2D imaging map.

Figure 6 shows the FTIR 2D imaging of pHEMA/PAA sponges obtained from SIPNs loaded with TEPA. Loading with the poly(ethylene amine) changes the refractive index of the gels, which show again strongly derivative-shaped C=O and C–O–C stretching bands. The most relevant feature is a composite band that shows two maxima at 1660 and 1610 cm^–1^. The latter is ascribed to the NH deformation of a primary amine in TEPA,^38^ while the first component can be assigned to the stretching vibration of the carboxylic groups in PAA, when they are neutralized by a poly(ethylene amine).^39^ Namely, the amine group of TEPA interacts with the acid sites of PAA, interfering with the interchain hydrogen bonds; the H atom of the carboxylic group is included in the amine groups, and the carboxylate vibration is shifted to a higher wavenumber with respect to the gels swollen in water solutions at the same pH. As shown by the 2D imaging maps, in the presence of Cu(II) ions, the peak is no longer clearly observable, all across the gel’s surface. Our hypothesis is that the carboxylate vibration is either shifted back to lower wavenumbers (convoluting with the TEPA NH deformation band) or its intensity is decreased, following the formation of a ternary polymer–metal complex by both PAA and TEPA with the copper ions. Kabanov et al.^40^ reported the formation of mixed Cu(II) complexes formed by PAA and poly(ethylene imines), where two coordination sites are covered by PAA carboxylates, and two by amine groups. A similar behavior might occur in the case of the PAA-Cu-TEPA complex.

**Figure 7:**
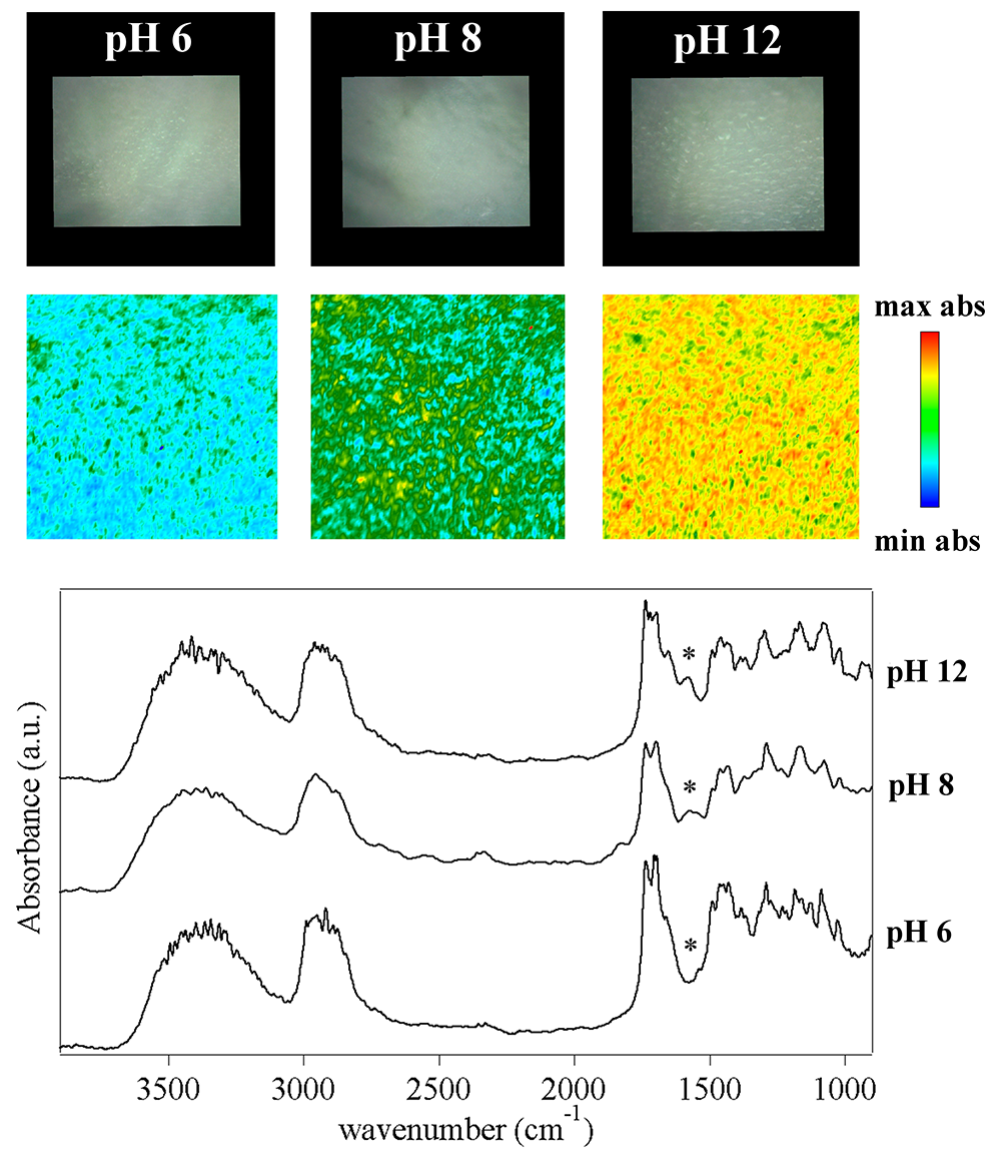
FTIR 2D imaging of pHEMA/PVP sponges obtained from hydrogels that were swollen with water at different pH values. Below each visible image, the corresponding 2D FTIR imaging map shows the intensity of the band at 1578 cm^–1^ (assigned to the stretching of enolate ions in PVP). All maps have dimensions of 700 × 700 μm^2^. The bottom panel shows representative spectra of pixels (5.5 × 5.5 μm^2^) in the corresponding 2D imaging map.

Figure 7 shows the FTIR 2D imaging of pHEMA/PVP sponges obtained from gels swollen in water at different pH values. All of the main absorptions of the two polymers are clearly observable;^36,41^ however, a band at 1578 cm^–1^ progressively emerges when the pH changes from 6 to 8 and 12, as evidenced in the 2D imaging maps. This band is assigned either to a combination of O–H and C–H bending^42^ or to the presence of an enolate ion, even if the latter would be expected at slightly higher wavenumbers. Indeed, as previously reported, the enolate form of the enol structure of PVP becomes more stable at pH ≫ 10.^28^ It must be noticed that the carbonyl band of the lactam form of PVP is also observable at 1655 cm^–1^.

**Figure 8:**
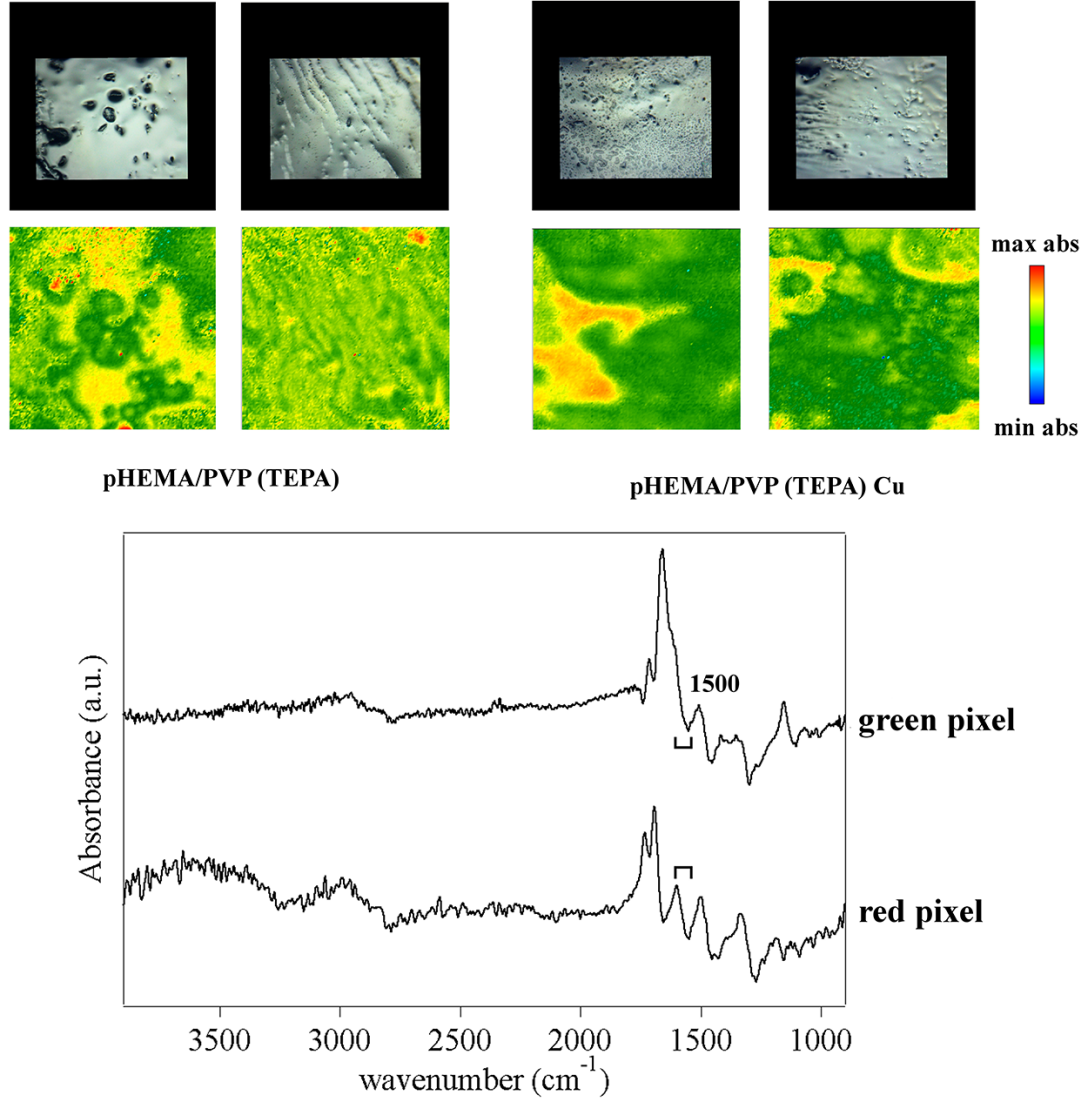
(Top, left panels) FTIR 2D imaging of sponges obtained from pHEMA/PVP hydrogels swollen with a TEPA solution (20%); (top, right panels) sponges obtained from gels that were swollen with the TEPA solution and then placed on a bronze coin mock-up containing Cu(II) corrosion products. Below each visible image, the corresponding 2D FTIR imaging map shows the intensity of the 1615–1535 cm^–1^ region. All maps have dimensions of 700 × 700 μm^2^. The bottom panel shows representative spectra of high (red) or low (green) intensity pixels (5.5 × 5.5 μm^2^) in the corresponding 2D Imaging map.

When TEPA is uploaded in the pHEMA/PVP gels, an inhomogeneous pattern is observed in the FTIR 2D Imaging maps of the absorbance intensity in the 1615–1535 cm^–1^ region (see Figure 8). In the main portion of the maps (yellow-red pixels), the NH deformation band of TEPA at 1600 cm^–1^ is clearly observable, as part of a derivative peak whose flexus falls at ca. 1580 cm^–1^. This band likely includes the contribution from enolate groups in PVP. In some portions (green pixels), no band around 1600 cm^–1^ is detected; instead, a shoulder to the PVP carbonyl peak is observed at ca. 1620 cm^–1^, along with a derivative band with a maximum at 1500 cm^–1^ and a flexus at ca. 1485 cm^–1^. We assigned the shoulder to the TEPA NH band (shifted upwards), and the derivative band to the enolate (shifted downwards) and CH_2_ vibrations. The band shifts were ascribed to interactions between the amine groups in TEPA and polar or charged CO groups in PVP. When Cu(II) ions are uploaded in the gel, the shifts are observed in significantly larger portions of the maps, which show a majority of green pixels. It is known that PVP is able to coordinate with copper through the O atom rather than N.^43^ Therefore, we hypothesized that the interactions between PVP and TEPA are favored by the coordination of Cu(II) with CO groups and amines, which come close together while binding to the ions.

**Figure 9:**
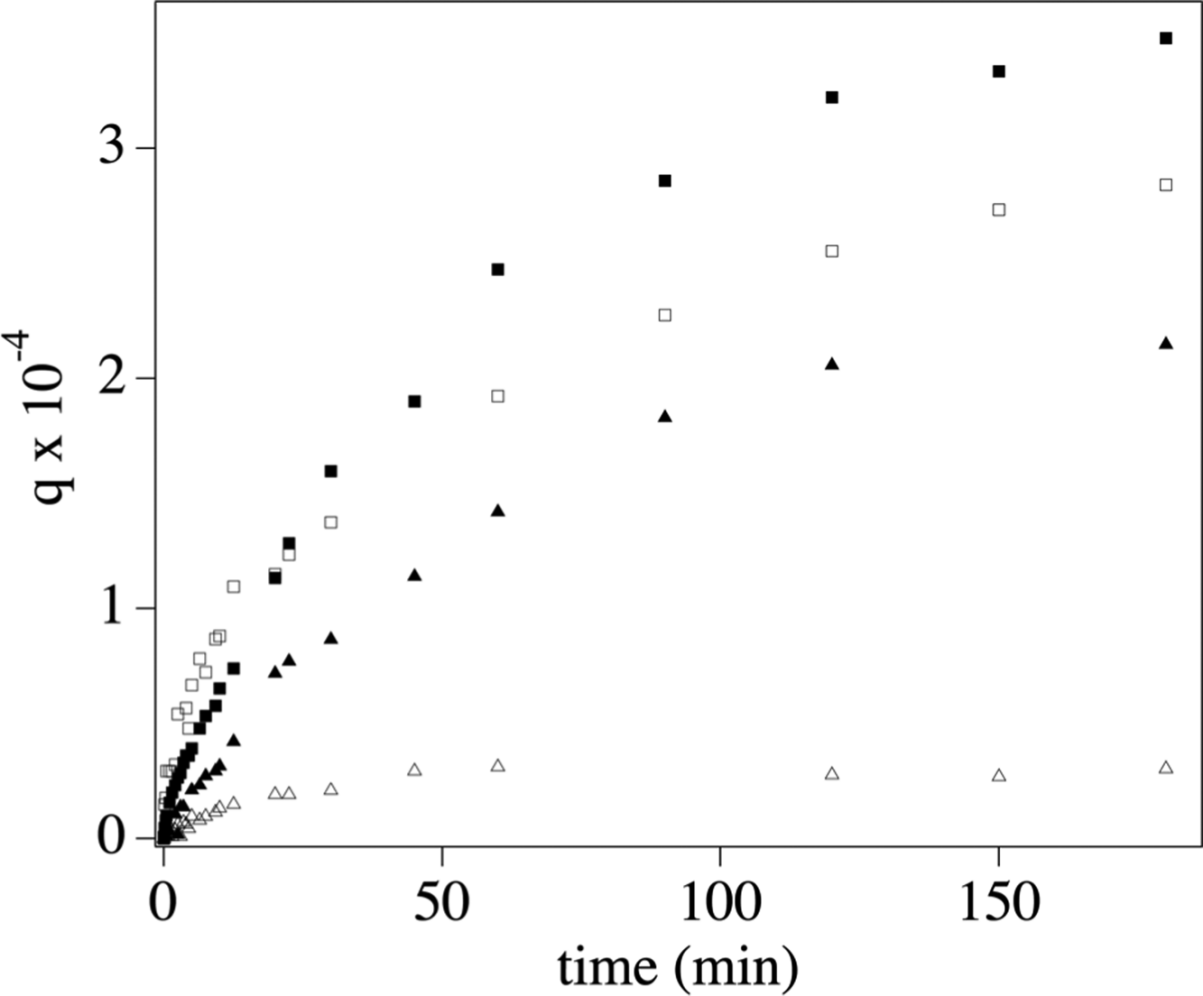
Plots of the adsorption efficiency *q* (grams of solute sorbed per gram of sorbent) over time for the uptake of Cu(II) ions by the pHEMA/PAA (squares) and pHEMA/PVP (triangles) SIPNs at pH 6 (empty markers) and 8 (full markers).

Adsorption kinetics highlighted the specific effect of the different SIPNs’ structures and functional groups on the uptake of Cu(II) ions. Figure 9 summarizes the trend of the adsorption efficiency, *q* (grams of solute sorbed per gram of sorbent), over time for the pHEMA/PAA and pHEMA/PVP SIPNs at pH 6 and 8. The values of *q* approaching the equilibrium value (*q*_e_) follow the trend PAA_pH 8 > PAA_pH 6 > PVP_pH 8 > PVP_pH 6. This confirms that the presence of carboxylate (in PAA) and enolate groups (in PVP) is a major driving force to the Cu(II) complexation by the SIPNs, and the effect of alkalinity on the increase of *q* is even more pronounced for PVP than PAA. Regarding the first stages of the adsorption kinetics, steeper curves are observed for pHEMA/PAA SIPNs, namely, pHEMA/PAA at pH 6 has the steepest initial increase.

The uptake curves of materials are traditionally fitted to different types of equations, i.e., diffusion-controlled models (intraparticle diffusion, IPD,^44,45^ diffusion-adsorption,^46^ adsorption dynamic intraparticle model,^47^ double-exponential^48^), transport models (Peppas equation or, alternatively, the Weibull function^49,50^), and adsorption-controlled kinetics. In the latter case, pseudo-first-order (*K*1), pseudo-second-order (*K*2), and Elovich models are generally proposed and critically compared. For instance, Azizian^51^ derived both *K*1 and *K*2 equations independently and concluded that the initial concentration of solute (*C*_0_) determines the kinetic regime, e.g., *K*1 provides the best fit when *C*_0_ is very high compared to the coverage of available sites in the sorbent, while *K*2 fits adsorption curves better when *C*_0_ is not too high with respect to the coverage. However, as reported in the literature,^52,53^ obtaining a good fit of the experimental data is not sufficient to validate the fitting model, as the underlying mechanism may be different: the literature reports several cases, for data sets well fitted by *K*2, where diffusion was recognized as the main contribution.^46,47,52,54−56^

Taking into account these considerations, we fitted the adsorption curves of the two SIPNs at pH 6 and 8 by different models.^52^ To obtain statistically relevant comparisons (i.e., which model provides better fits), we used the fitting models on the original scale (*y* = *q*(*t*)) rather than adopting transformed scales or linearized equations.^52,57,58^ The details on the modeling (i.e., analytical equations, full set of fitted curves, and extracted parameters as rate constants *k*, *q*_e_, and chi-square) are reported in the SI file (Tables S5–S8 and Figures S7–S10).

**Figure 10:**
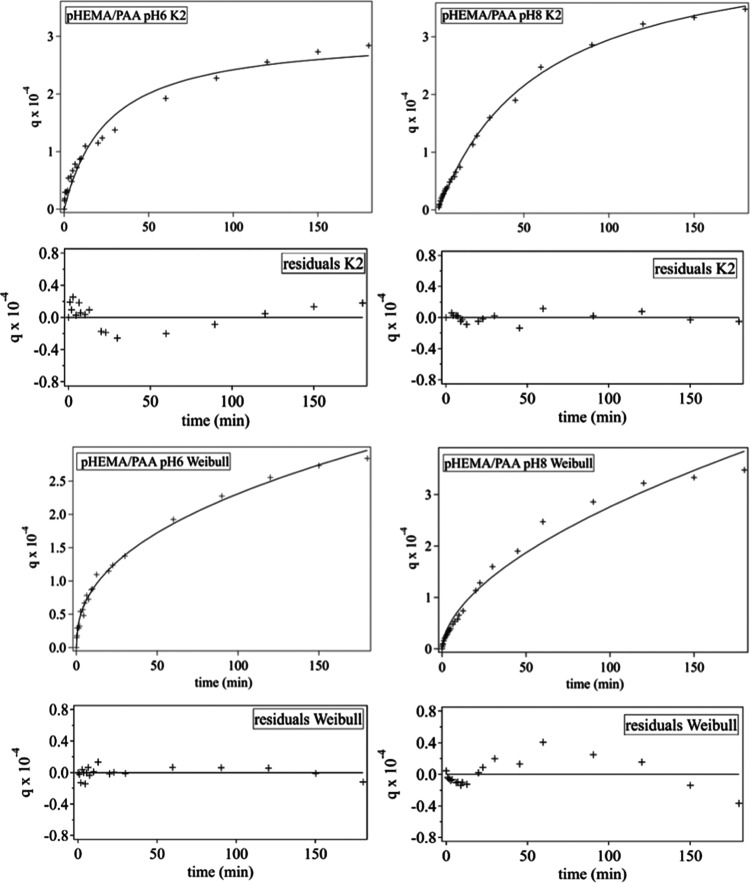
Evolution of Cu(II) adsorption efficiency for pHEMA/PAA SIPNs at pH 6 and 8. The uptake curves (0–180 min) are fitted to the pseudo-second-order kinetic model (*K*2) and Weibull equation. The bottom panels show the residuals of the *K*2 and Weibull fittings.

In the case of pHEMA/PAA SIPNs at pH 6, the pseudo-second-order model provides a better fit than the pseudo-first-order model (see Figure S7; the *K*2 fit is shown in Figure 10), even though both *K*1 and *K*2 underestimate the uptake in the first part of the process. As recently reported by Simonin, this behavior suggests a two-step process where the fast initial adsorption (diffusion-limited) driven by capillary forces is followed by a slower uptake as a consequence of the diffusion of the solute in the smaller pores or of slow adsorption (e.g., at less available sites).^52^ The presence of a two-step mechanism is also confirmed by the good fit of the uptake using the double-exponential equation (see Figure S7). Both the IPD and Weibull models provide very good fits of the uptake (the value of *n*_W_ ∼ 0.4 from the Weibull fit indicates Fickian transport), even though they do not account well for the uptake decrease in the very final stages (see Figures 10 and S7), supporting the hypothesis that the uptake process in the pHEMA/PAA at pH 6 is largely controlled by diffusion.

At pH 8, the IPD and Weibull equations provide worse fits, and overall, the pseudo-second-order model provides a better fit than the pseudo-first-order model (see Figures 10 and S8), i.e., the contribution of diffusion seems to be less significant than at pH 6; this is in good agreement with the much higher porosity exhibited by the pHEMA/PAA gel after carboxylic groups are deprotonated.

**Figure 11:**
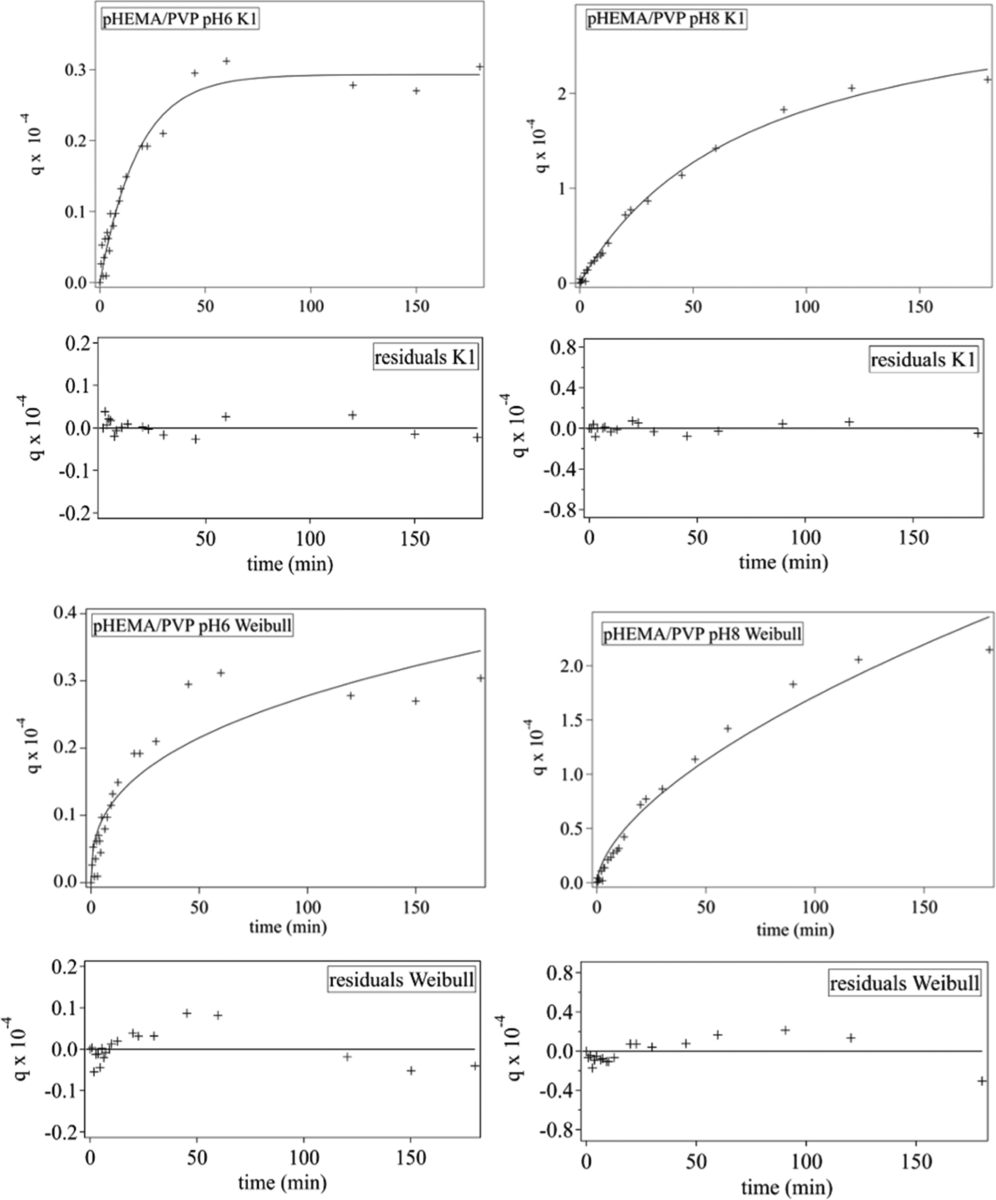
Evolution of adsorption efficiency for the uptake of Cu(II) ions by the pHEMA/PVP SIPNs at pH 6 and 8. The uptake curves (0–180 min) are fitted to the pseudo-first-order kinetic model (*K*1) and Weibull equation. The bottom panels show the residuals of the *K*1 and Weibull fittings.

For pHEMA/PVP SIPNs, in general, the pseudo-first-order model provides a better fit than the pseudo-second-order model at pH 6 and 8 (see Figures S9 and S10; the *K*1 fit is shown in Figure 11). The IPD and Weibull models do not fit well the data (see Figures 11, S9, and S10), nor does the double-exponential equation, while a single-exponential provides better fits (see Figures S9 and S10). This suggests that the process involves a single main step, probably controlled by adsorption rather than diffusion, which is reasonable considering the porogen role of PVP in the SIPN network.

Finally, no satisfactory fittings could be obtained with the Elovich model of chemisorption.

**Figure 12:**
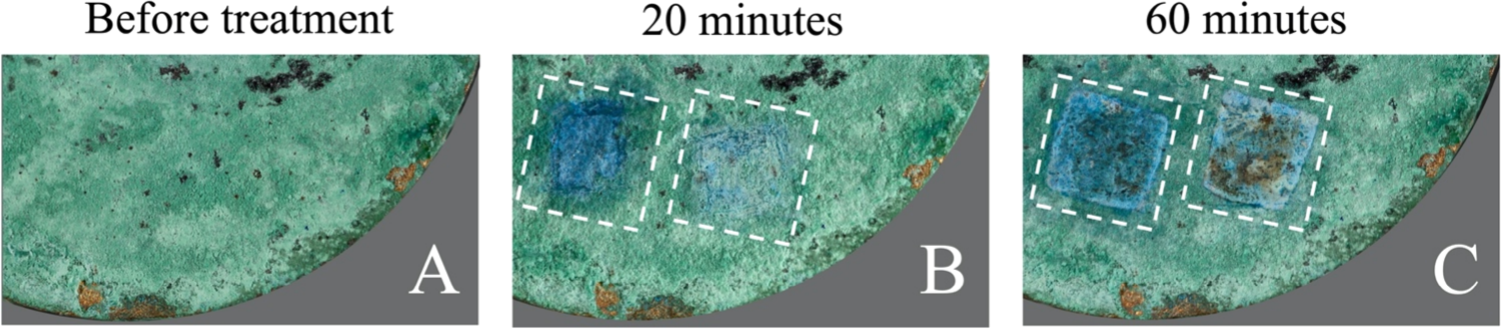
Artificially aged bronze coin before (A) and after 20 (B) and 60 min (C) of cleaning with pHEMA/PVP (left dashed box) and /PAA (right dashed box) SIPNs loaded with an aqueous TEPA solution (20% w/w). The application of the pHEMA/PAA gel led to the progressive removal of the green corrosion products (copper oxychlorides), preserving the red cuprite layer that is inhomogeneously present on the coin surface, with overall higher effectiveness than its /PVP counterpart.

**Figure 13:**
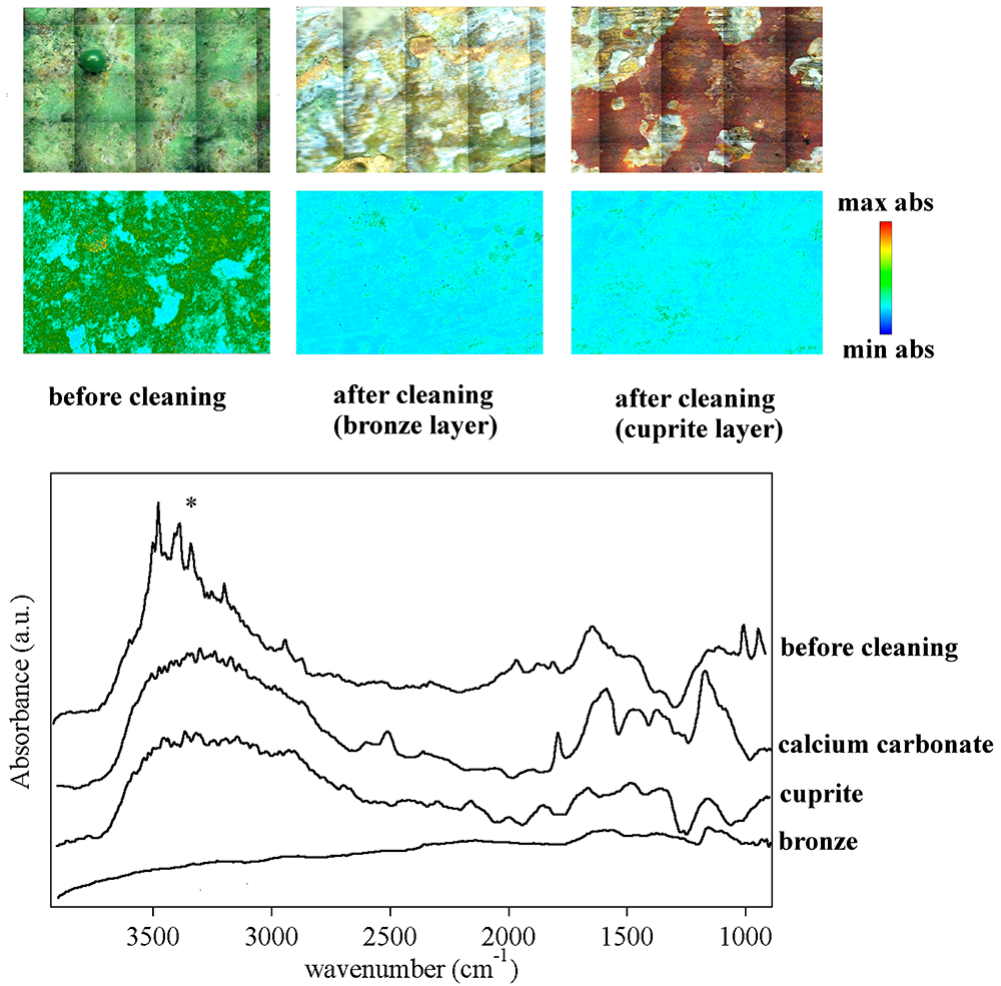
(Top) Optical micrographs and 2D FTIR imaging, detailing the cleaning areas shown in Figure 12. (Left panel) Artificially aged bronze coin before cleaning. (Center and right panels) Coin surface after cleaning. Below each micrograph, the corresponding 2D FTIR imaging map shows the localization of atacamite and paratacamite residues (intensity of the band between 3550 and 3300 cm^–1^ corresponding to the stretching of OH groups in the copper oxychlorides species). All maps have dimensions of 1400 × 2000 μm^2^. The bottom panel shows representative spectra of pixels (5.5 × 5.5 μm^2^) in the corresponding 2D imaging maps for cleaned bronze surface, red layer of cuprite, calcium carbonate patinas, and copper oxychlorides (atacamite, paratacamite).

**Figure 14:**
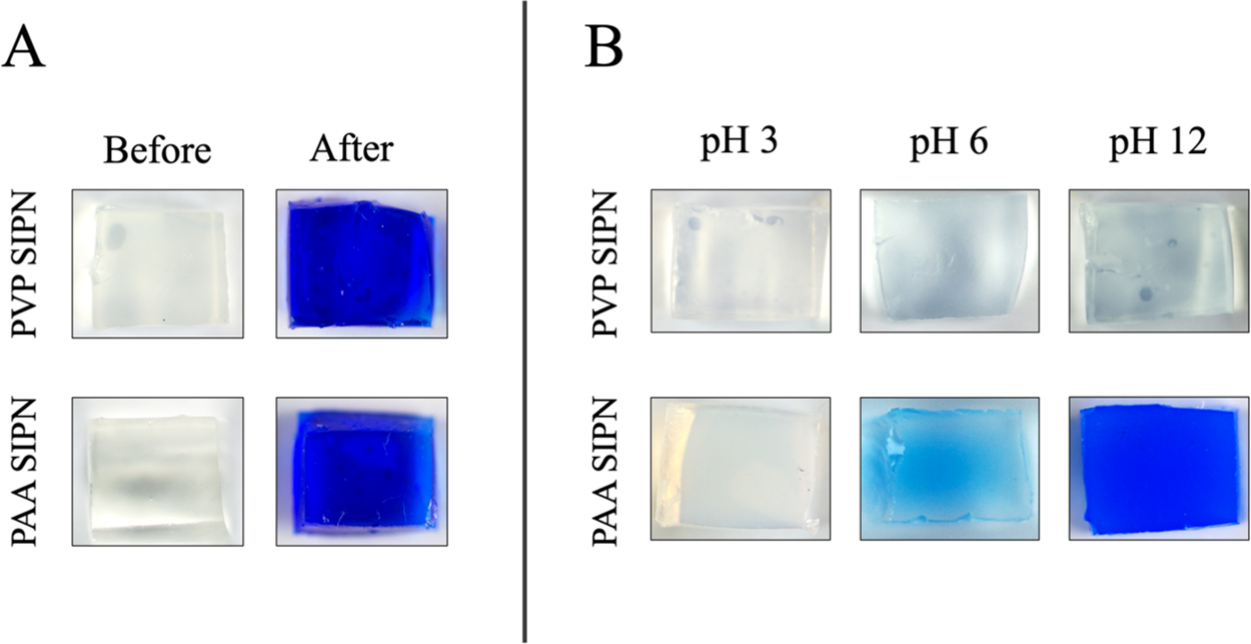
(A) Gel surfaces before and after the application on the corroded bronze coin. The strong blue discoloration due to the formation of Cu(II)-TEPA complexes is clearly visible. (B) TEPA-loaded SIPNs after application on corroded bronze coins, and successive equilibration in aqueous solutions at different pH (immersion time: 3 h). The blue color is due to the presence of the Cu(II)–TEPA complex formed upon the removal of copper corrosion layers. The pHEMA/PVP SIPN does not retain the complex over the full pH range, and becomes transparent. Instead, the release of the complex by the pHEMA/PAA can be controlled by tuning pH: complete release at pH 3; partial release at pH 6; while at pH 12, the gel retains the blue complex.

Overall, the analysis of the curves confirmed the importance of pH control to boost the uptake of Cu(II) ions and indicated that the ion–matrix interaction is stronger for pHEMA/PAA than pHEMA/PVP. Accordingly, we expected an increased efficacy in the removal of copper corrosion layers by the network interpenetrated with PAA with respect to PVP. Figures 12 and 13 show the application of SIPNs loaded with a 20% (w/w) aqueous solution of TEPA on the surface of a bronze coin that was artificially aged to mimic archaeological bronze artifacts. After aging, the coin surface is covered with a thick and heterogeneous green patina of copper oxychlorides, which show characteristic IR bands between 3550 and 3300 cm^–1^ (OH stretching) and at 950 cm^–1^ ^59^ (see Figures 12A and 13). As the gels absorb Cu(II) ions from the corrosion layers, they become strongly blue-colored owing to the formation of the Cu(II)–TEPA complex (see Figure 14A). In principle, the color change might allow following the cleaning process, indicating the time when a copper-saturated gel needs to be refreshed.

A significant difference in the performance of the two SIPNs was highlighted already after 20 min of application (see Figure 12B): the pHEMA/PAA gel thinned the corrosion patina without excessive release of the chelating solution, showing higher effectiveness than the /PVP SIPN, which we explained by hypothesizing a higher affinity of the complex to PAA, in good agreement with the stronger ion–PAA matrix interaction discussed above. This hypothesis was also supported by the pH-responsive behavior of the gels, as shown in Figure 14B: the pHEMA/PAA gels retain the Cu(II)–TEPA complex even when they are exposed to an aqueous solution at pH 12 (where the carboxylates likely participate in the formation of stable ternary complexes). Instead, the pHEMA/PVP gels exchange completely with highly alkaline water.

Over 60 min (Figures 12C and 13), the application of the pHEMA/PAA gel led to the progressive removal of the oxychlorides, preserving the inner red layer (whose color is indicative of cuprite, Cu_2_O^60^) inhomogeneously present over the surface; such controlled cleaning action is crucial since cuprite is a protective layer that passivates the surface against further corrosion. Where cuprite was not present, the cleaning intervention brought back the bronze surface or whitish patinas of calcium carbonate (identified thanks to characteristic IR bands at 2512, 1793, and 1463 cm^–1^ ^61^) that can eventually be removed using a complexing agent selective for calcium. 2D FTIR imaging confirmed the removal of copper oxychlorides at the micron scale (see Figure 13), down to the detection limit of the instrument (<1 pg/pixel; 1 pixel = 5.5 × 5.5 μm^2^ ^62^).

The TEPA-loaded pHEMA/PVP and /PAA gels can be reused several times after removal of corrosion layers by exchanging with water, and then uploading fresh TEPA solution. However, while the pHEMA/PVP gels exchange completely in a few hours under all pH conditions, the pHEMA/PAA show a pH-responsive behavior as carboxylates turn back into weakly binding carboxylic groups (see Figure 14B). For instance, equilibrating the gels at pH 3 for a few hours results in the quantitative exchange of the complex solution with water, while the process occurs overnight at pH 6. This makes the new pHEMA/PAA SIPNs appealing to a wide range of application, even beyond Cultural Heritage preservation, where controlled uptake and/or release of molecules is needed (e.g., drug delivery,^63−65^ wastewater treatment,^66^ agricultural industry,^67−69^ and food chemistry).^70^

## Conclusions

A novel pHEMA/PAA SIPN was formulated and applied here, for the first time, to achieve enhanced capture of copper ions and remove corrosion layers, improving on existing pHEMA/PVP networks by taking advantage of the chelating ability of pH-responsive carboxylic groups in PAA.

The microstructure and sorption properties of the SIPNs were investigated as a function of pH. In the case of pHEMA/PAA SIPNs, carboxyl groups in the PAA chains are progressively ionized with alkalinity as confirmed by FTIR spectroscopy. This results in the swelling of the polymer network as evidenced by an increase in the porosity at the macro- and nanoscales, and in the equilibrium water content. TEPA molecules interact with carboxyls in PAA, making the SIPN more compact by screening the repulsion between carboxylate groups. When Cu(II) ions are absorbed in the TEPA-loaded network, ternary PAA-Cu-TEPA complexes are probably formed, where two coordination sites are covered by PAA carboxylates, and two by amine groups. In the case of pHEMA/PVP SIPNs, the pH increase progressively leads to the conversion of the pyrrolidone ring in its enol tautomer. Significant changes in the macroporosity are observed passing from 6 to 12, with a neat decrease in pores size when pH 12 is reached. The porosity at the nanoscale decreases at pH 12, as the inter- and intramolecular hydrogen bonds between enol and enolate groups in the PVP chains likely cause a decrease in the mesh size. FTIR seemed to evidence that the interactions between PVP and TEPA are favored by the formation of ternary complexes with Cu(II) ions, where CO and amine groups come closer while binding to the metal.

For the same pH values, the interaction of Cu(II) ions with the gel matrix is stronger in the case of pHEMA/PAA, leading to a higher amount of ions sorbed compared to pHEMA/PVP. For the pHEMA/PAA gels, the uptake process is likely constituted by a fast initial adsorption stage (diffusion-limited) of the ions driven by capillary forces, followed by a second stage controlled by the diffusion in smaller pores and adsorption at less available sites. As expected, the contribution of diffusion is more significant at pH 6, while at higher pH values, the deprotonation of carboxyl groups increases the porosity at the macro- and nanoscales, favoring the diffusion of ions. For the pHEMA/PVP gels, the uptake process is probably controlled by adsorption, rather than diffusion, as expected considering the porogen role of PVP in the network.

When the TEPA-loaded gels are applied onto corroded bronze coins, they gradually release the polyamine solution on the surface, solubilizing and removing the Cu(II) oxychlorides in the corrosion layers. The dissolved copper ions migrate into the gels and form complexes, which gives the gels an intense blue color. Notably, the pHEMA/PAA SIPN exhibited significantly better performances than its /PVP counterpart, allowing the removal of oxychlorides more effectively and in shorter times. The inner red corrosion product (cuprite), underlying the oxychloride layers, was preserved by the selective cleaning action of the SIPNs, which is beneficial since cuprite is normally left to passivate the surface of bronze against recurring corrosion. The gels can be reused several times by exchanging with water and uploading fresh TEPA solution. In addition, the pHEMA/PAA exhibits a marked pH-responsive behavior, where simply tuning pH, it is possible to completely release the Cu(II)–TEPA complex in a few hours (pH 3) or overnight (pH 6), or retain the complex at alkaline pH (12).

Overall, pHEMA/PAA SIPNs are used as new promising tools for the capture of copper ions and the removal of corrosion from metallic surfaces, and their pH-responsive character opens possible applications in transversal fields, even beyond Cultural Heritage conservation, such as drug delivery, wastewater treatment, agricultural industry, and food chemistry.

## Supporting Information Available

Binarized SEM images of sponges used for chord length distribution analysis; cumulative frequencies of chords; slopes and corresponding persistence length of pore size and wall size distributions; DSC thermograms of the hydrogels, DSC and TGA data and additional comments; description of kinetic models and fitting parameters for the different models applied to the uptake of Cu(II) ions by the hydrogels at different pH values (PDF)
