# Supplementary material for: pH-Responsive Semi-Interpenetrated Polymer Networks of pHEMA/PAA for the Capture of Copper Ions and Corrosion Removal
Source: ACS Appl Mater Interfaces. 2022 Jan 28;14(5):7471–85. doi: 10.1021/acsami.1c22837 (PMC8832396; doi:10.1021/acsami.1c22837)
Supplement: Supplementary file 1 — am1c22837_si_001.pdf [file am1c22837_si_001.pdf]

Supporting Information for

PH-RESPONSIVE SEMI-INTERPENETRATED POLYMER NETWORKS OF PHEMA/PAA FOR THE CAPTURE OF COPPER IONS AND CORROSION REMOVAL

Teresa Guaragnone‡, Marta Rossi‡, David Chelazzi, Rosangela Mastrangelo, Mirko Severi,

Emiliano Fratini, Piero Baglioni**

Department of Chemistry "Ugo Schiff" and CSGI, University of Florence, via della Lastruccia 3-

Sesto Fiorentino, I-50019, Florence, Italy.

*email: piero.baglioni@unifi.it; emiliano.fratini@unifi.it

‡ These authors contributed equally

Chord length distribution analysis

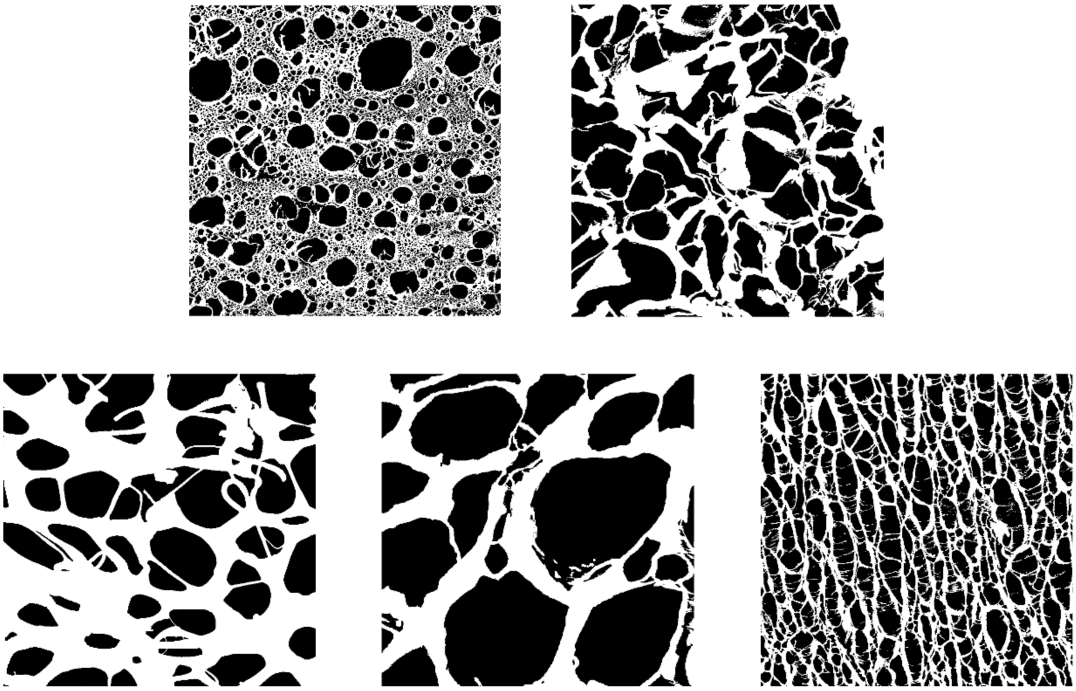

Figure S1. Binarized SEM images of pHEMA/PAA and pHEMA/PVP gels, used to implement the chord length distribution. Top row, from left to right: pHEMA/PAA at pH 8; pHEMA/PAA at pH 12. Bottom row, from left to right: pHEMA/PVP at pH 6; pHEMA/PVP at pH 8; pHEMA/PVP at pH 12. Magnification and scale are the same as Figure 1 in the main text.

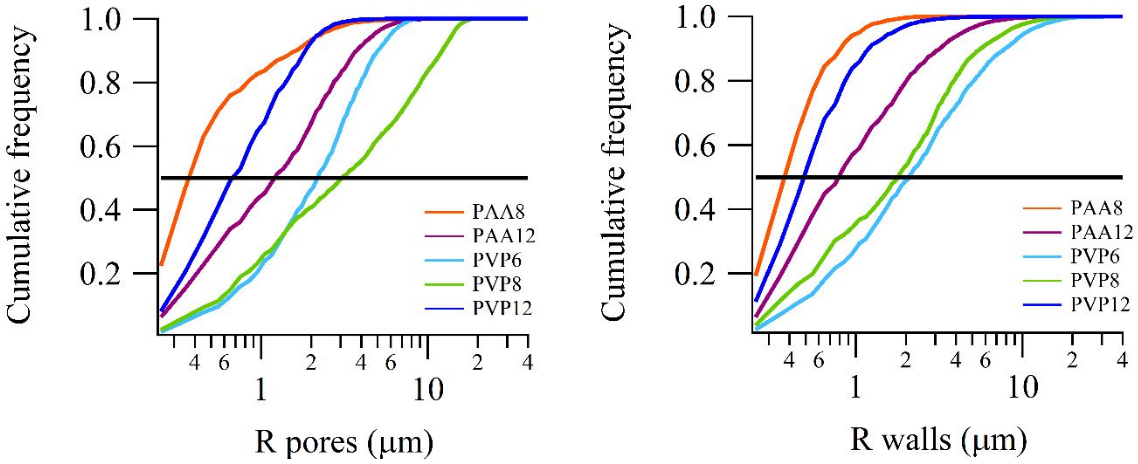

Figure S2. Cumulative frequencies of chords, obtained from chord analysis on the pores (left) and the walls (right) of the binarized SEM images (figure S1). The black lines indicate the R value at 50% of the total population.

Table S1. Slopes (1/λ) and corresponding persistence length (λ) of pores size distributions of pHEMA/PAA and pHEMA/PVP gels at different pH values, fitted to equation 3 (see main text). Uncertainties on slopes account for the 10% at least of λ values. In some cases, two trends were evident in the distributions, at low and high ranges of the chord length (R). The last columns show: the average diameter D with standard deviation, calculated with the software Image J on the same images and on three images at different magnification (values in brackets); the value of R at 50% of the total population, obtained from data in figure S2.

Sample	Slope @low R (1/λ)	λ @low R (μm)	Slope @high R (1/λ)	λ @high R (μm)	Average λ (μm)	Average D (Image J, μm)	R at 50% (Cumulant frequency)
pHEMA/PAA, pH 8	5.3 ± 0.4	0.19 ± 0.02	1.1 ± 0.1	0.88 ± 0.09	0.5 ± 0.5	0.4 ± 0.4 (2.5 ± 1.5)	0.40 (±0.05)
pHEMA/PAA, pH 12	1.6 ± 0.1	0.64 ± 0.06	0.65 ± 0.02	1.5 ± 0.1	1.1 ± 0.6	1.34 ± 1.5 (1.0 ± 2.4)	1.20 (±0.05)
pHEMA/PVP, pH 6	--	--	0.27 ± 0.02	3.7 ± 0.4	--	2.61 ± 1.8 (2.8 ± 1.0)	2.20 (±0.05)
pHEMA/PVP, pH 8	0.51 ± 0.08	1.9 ± 0.3	0.19 ± 0.03	5.4 ± 0.9	3.7 ± 2.4	3.11 ± 4.0 (2.3 ± 1.6)	3.15 (±0.05)
pHEMA/PVP, pH 12	--	--	1.2 ± 0.1	0.80 ± 0.08	--	1.04 ± 0.9 (0.9 ± 1.9)	0.70 (±0.05)

Table S2. Slopes (1/λ) and corresponding persistence length (λ) of gels' walls size distributions of pHEMA/PAA and pHEMA/PVP gels at different pH values, fitted to equation 4 (see main text). Uncertainties on slopes account for the 10% at least of λ values. The last column shows the value of R at 50% of the total population, obtained from data in figure S2.

Sample	Slope (1/λ)	λ (μm)	R at 50% (Cumulant frequency)
pHEMA/PAA, pH 8	3.2 ± 0.1	0.31 ± 0.03	0.40 (±0.05)

pHEMA/PAA, pH 12	0.85 ± 0.03	1.2 ± 0.1	0.80 (±0.05)
pHEMA/PVP, pH 6	0.40 ± 0.02	2.5 ± 0.2	2.10 (±0.05)
pHEMA/PVP, pH 8	0.43 ± 0.03	2.3 ± 0.2	1.80 (±0.05)
pHEMA/PVP, pH 12	2.4 ± 0.1	0.42 ± 0.04	0.50 (±0.05)

Thermal analyses

DSC thermograms

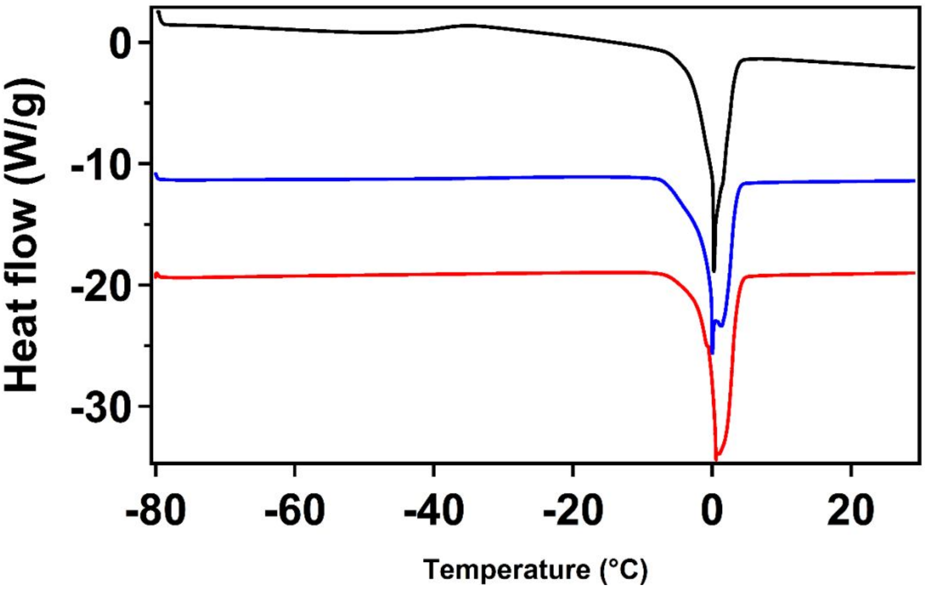

Figure S3. DSC thermograms for the pHEMA/PAA semi-IPNs gels swollen at pH 6 (black line), pH8 (blue line) and pH12 (red line). All thermograms were normalized to sample weight. The reported curves are offset along y-axis for clarity.

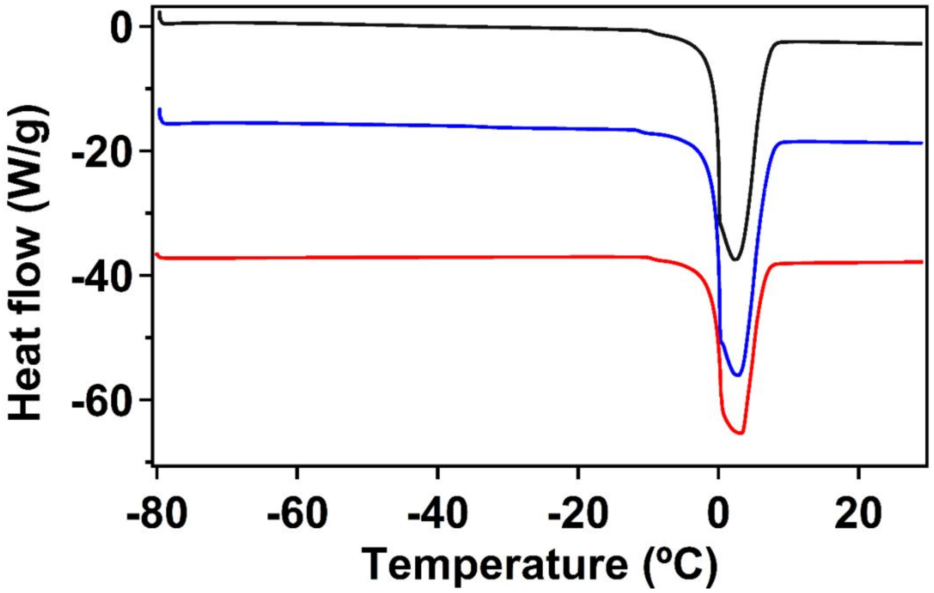

Figure S4. DSC thermograms for the pHEMA/PVP semi-IPNs gels swollen at pH 6 (red line), pH8 (black line) and pH12 (red line). All thermograms were normalized to sample weight. The reported curves are offset along y-axis for clarity.

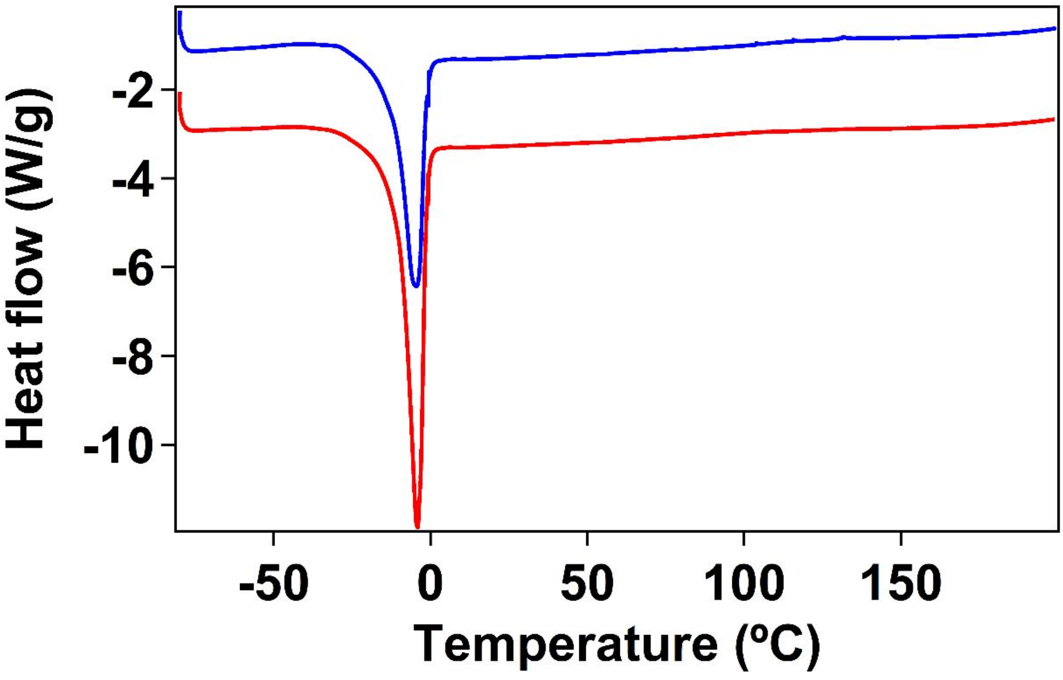

Figure S5. DSC thermograms for the pHEMA/PAA semi-IPNs gels swollen in a water solution of TEPA (20% w/w) (red line) and after the absorption of Cu(II) (blue line). Both thermograms were normalized to sample weight. The reported curves are offset along y-axis for clarity.

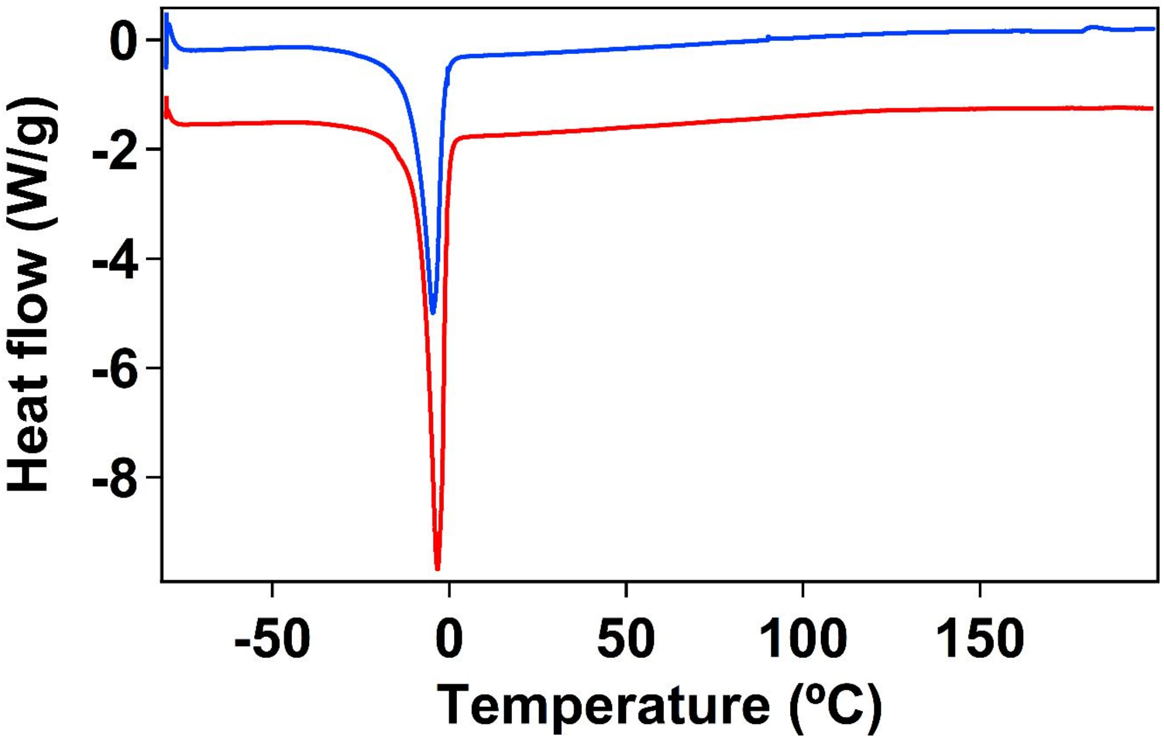

Figure S6. DSC thermograms for the pHEMA/PVP semi-IPNs gels swollen in a water solution of TEPA (20% w/w) (red line) and after the absorption of Cu(II) (blue line). Both thermograms were normalized to sample weight. The reported curves are offset along y-axis for clarity.

The study of the amount and types of water loaded in the hydrogels provided information on the absorption and permeation properties of these systems. DSC curves of all the gels are reported in Figure S3-6. The EWC in pHEMA/PAA SIPNs, swollen at different pH, is mainly due to the gels' porosity and the hydrophilic character of polymer matrix (mostly PAA). At pH 6, where the carboxylic group are completely protonated, the EWC is about 43% (see Table S3), only slightly higher than in the case of classical pHEMA chemical gels (i.e. 38%) [1] [2], and it increases up to 57 and 79% at pH 8 and 12, owing to the ionization of the carboxyl groups and consequent pores enlargement. When the SIPN is loaded with TEPA (pH 12), the solvent content is ca. 76%. After the absorption of Cu(II) this value decreases to 71%, indicating that some free water is lost through evaporation during the application of the gel on the bronze coin, despite having covered the gel with parafilm. The FWI values increase in passing from pH 6 to 12 (see Table S3), consistently with the presence of more hydrophilic moieties in the network (carboxylate groups in PAA), and with a higher meso- and macroporosity as shown by SAXS measurements and by the chord analysis implemented on SEM images.

Table S3. Data obtained from DSC and TGA analysis of pHEMA/PAA SIPNs swollen in water at pH 6, 8, and 12 and in TEPA water solution before and after Cu(II) uptake.

	pH 6	pH 8	pH 12	TEPA	TEPA Cu
ΔH_{tr} (J/g)	76.5 ± 1.7	154.3 ± 1.27	293.2 ± 6.1	127.4 ± 4.1	109.0 ± 3.1
EWC	42.9% ± 0.9%	56.8% ± 1.1%	79.1 % ± 3.7%	76.3% ± 1.6%	71.3% ± 1.4%
FWI	0.53 ± 0.02	0.81 ± 0.02	1.11 ± 0.07	0.50 ± 0.03	0.46 ± 0.02

Both the EWC and FWI of the pHEMA/PVP SIPNs remain unchanged when the gels are swollen in water at different pH (Table S4). In any case, these parameters have higher values than those of pHEMA/PAA gels, which is explained considering the high relative content of PVP, a highly hydrophilic polymer. The EWC does not change significantly in the presence of TEPA and Cu(II) ions, demonstrating the high hydrophilicity of these systems.

It must be noticed that for both systems there is a significant decrease in the heat of the melting transition (ΔH_{tr}, see Table S3-4) when the gels are uploaded with the TEPA solution, and when Cu(II) ions are absorbed in the gels. The FWI decreases accordingly. In the case of TEPA-loaded gels, this was ascribed to strong hydrogen bonding taking place between the amine and water molecules, which

also explains the lower critical solution temperature (as previously observed in amine-water solutions). When the gels absorb the copper ions, the further decrease was explained considering that part of the bulk water molecules coordinate with the metal ions, participating in the formation of complexes.

Table S4. Data obtained from DSC and TGA analysis of pHEMA/PVP SIPNs swollen in water at pH 6, 8, and 12 and in TEPA water solution before and after Cu(II) uptake.

	pH 6	pH 8	pH 12	TEPA	TEPA Cu
ΔH_{tr} (J/g)	296.1 ± 3.9	317.2 ± 1.1	299.5 ± 5.26	154.6 ± 2.5	133.5 ± 4.1
EWC	88.3% ± 0.5%	89.1% ± 1.3%	89.0% ± 2.1%	86.5% ± 0.01%	85.6% ± 0.8%
FWI	1.01 ± 0.02	1.07 ± 0.02	1.01 ± 0.04	0.54 ± 0.01	0.47 ± 0.02

Kinetic Models

Pseudo-first order rate law, K1

The pseudo-first order kinetic equation, initially introduced by Lagergren [3], is generally used in the form proposed by Ho and McKay [4]:

$$\ln\left[q_e - q(t)\right] = ln q_e - k_1 t$$

where q is the amount of adsorbed solute, q_e its value at equilibrium, k_1 is the pseudo-first order rate constant, and t is the time.
For fitting the experimental data, we used the alternative expression:

$$q(t) = q_e\,[1 - \exp\,(-k_1 t)].$$

The pseudo-first order kinetic equation differs in principle from a true first order equation in two aspects [5] :

- q_e can, in principle, differ from the theoretical maximum adsorption capacity of the surface, since reacting surfaces can be inhomogeneous, and effects of transport phenomena and chemical reactions are often experimentally inseparable. Therefore, the parameter $k_1(q_e-q_t)$ does not represent the number of available sites, as opposed to true first order equations.
- The coefficient of the exponential term $exp(-k_1t)$ can be adjusted to values greater than 1, whereas it has to be strictly 1 in true first order equations.

Pseudo-second order rate law, K2

The pseudo-second order model assumes that the rate of adsorption of solute is proportional to the available sites of the adsorbent. The kinetics equation is normally used as indicated by Ho and McKay [4]:

$$\frac{t}{q(t)} = \frac{t}{q_e} + \frac{1}{k_2 {q_e}^2}$$

where k_2 is the pseudo-second order kinetic rate constant. q_e can, in principle, differ from the theoretical maximum adsorption capacity of the surface (see above), thus the model equation differs from a true second order equation.

To obtain statistically relevant comparisons with the other models (i.e. which one provides better fits), we used the following expression that considers the original scale (y = q(t)):

$$q(t) = q_e \frac{k_2^* t}{1 + k_2^* t}$$

where $k^*_2 = k_2 q_e$.

Intraparticle diffusion model, IPD

The intraparticle diffusion model employs a power law where $q \propto t^{1/2}$ [6]:

$$q(t) = k_{ipd} \cdot t^{1/2} + \mathrm{c}$$

where k_{ipd} is a rate constant and c is the thickness of a boundary layer on the adsorbent's surface.

Weibull function

The Weibull function is an empirical equation frequently applied to the analysis of dissolution and release studies:

$$q(t) = 1 - \exp\left(-k_w \cdot t^{n_w}\right)$$

where k_w defines the time scale of the process (i.e., the time required for the absorbate to cross the free path in the sorbent), and nw is used to indicate the transport mechanism: nw ≤ 0.75 corresponds to a Fickian transport, 0.75 < nw < 1 indicates a combination of a Fickian diffusion and a Case-II transport, nw>1 indicates a complex transport mechanism [7]. The Weibull function is commonly used as an alternative to the semi-empirical Korsmeyer-Peppas equation ($q(t) = kt^n$), which is in turn an extension of the Higuchi law ($q(t) = kt^{1/2}$) [7].

Double exponential model, DE

This is an empirical model that is not based on any assumption regarding the chemistry of the process but rather describes sorption from a mathematical point of view.
The model uses a double exponential function to correlate the two-step kinetics of the adsorption of a metal ion onto a matrix [8]:

$$q(t) = q_e - \frac{D1}{m}\exp\left(-K_{D1}t\right) - \frac{D2}{m}\exp\left(-K_{D2}t\right)$$

where D1 and D2 are sorption rate parameters (mmol L^{-1}) of the rapid and the slow step, respectively, and K_{D1} and K_{D2} are parameters (min^{-1}) controlling the mechanism; m is the adsorbent amount in the solution (g L^{-1}). If the exponential term corresponding to the rapid process is assumed to be negligible on the overall kinetics ($K_{D1}>>K_{D2}$), the model equation can be simplified to a single exponential (SE):

$$q(t) = q_e - \frac{D1}{m}\exp(-K_{D1}t)$$

Table S5. Fitting parameters and chi square (chi-sq) values for K1, K2, IPD, Weibull and DE equations applied to the uptake of Cu(II) ions by the pHEMA/PAA semi-IPN at pH 6.

	K1	K2	IPD	Weibull	DE
q_e	2.57 x 10^{-4} ± 0.11 x 10^{-4}	3.04 x 10^{-4} ± 0.14 x 10^{-4}	-	-	3.17 x 10^{-4} ± 0.17 x 10^{-4}
k_2^*	-	3.87 x 10^{-2} ± 0.48 x 10^{-2}	-	-	-
k_1	3.50 x 10^{-2} ± 0.42 x 10^{-2}	-	-	-	-
K_{D1}	-	-	-	-	1.14 x 10^{-2} ± 0.20 x 10^{-2}
K_{D2}	-	-	-	-	0.24 ± 0.07
k_w	-	-	-	3.29 x 10^{-5} ± 0.13 x 10^{-5}	-
n_w	-	-	-	0.42 ± 9.21 x 10^{-3}	-
D1	-	-	-	-	2.50 x 10^{-4} ± 0.12 x 10^{-4}
D2	-	-	-	-	5.66 x 10^{-5} ± 0.85 x 10^{-5}
k_{ipd}	-	-	2.18 x 10^{-5} ± 0.05 x 10^{-5}	-	-
c	-	-	1.30 x 10^{-5} ± 0.29 x 10^{-5}	-	-
chi-sq	9.77 x 10^{-9}	5.66 x 10^{-9}	2.10 x 10^{-9}	1.04 x 10^{-9}	7.99 x 10^{-10}

Table S6. Fitting parameters and chi square (chi-sq) values for K1, K2, IPD, Weibull and DE equations applied to the uptake of Cu(II) ions by the pHEMA/PAA semi-IPN at pH 8.

	K1	K2	IPD	Weibull	DE
q_e	3.52 x 10^{-4} ± 0.60 x 10^{-4}	4.68 x 10^{-4} ± 0.10 x 10^{-4}	-	-	3.62 x 10^{-4} ± 0.42 x 10^{-6}
k_2^*	-	1.70 x 10^{-2} ± 0.81 x 10^{-2}	-	-	-
k_1	1.97 x 10^{-2} ± 0.76 x 10^{-2}	-	-	-	-
K_{D1}	-	-	-	-	1.74 x 10^{-2} x 0.55 x 10^{-5}
K_{D2}	-	-	-	-	2.47 ± 3.59
k_w	-	-	-	2.07 x 10^{-5} ± 0.21 x 10^{-5}	-
n_w	-	-	-	0.56 ± 2.21 x 10^{-2}	-
D1	-	-	-	-	3.52 x 10^{-4} ± 0.04 x 10^{-4}

D2	-	-	-	-	$1.07 \times 10^{-5} \times 1.02 \times 10^{-5}$
k_{ipd}	-	-	$2.72 \times 10^{-5} \pm 0.10 \times 10^{-5}$	-	-
c	-	-	$-4.00 \times 10^{-21} \pm 5.58 \times 10^{-6}$	-	-
chi-sq	1.25×10^{-9}	9.93×10^{-10}	8.59×10^{-9}	6.16×10^{-9}	3.62×10^{-10}

Table S7. Fitting parameters and chi square (chi-sq) values for K1, K2, IPD, Weibull and DE equations applied to the uptake of Cu(II) ions by the pHEMA/PVP semi-IPN at pH 6. In this case, only the reduced DE equation (i.e. single exponential) provided good fits of the experimental data.

	K1	K2	IPD	Weibull	DE*
q_e	$2.93 \times 10^{-5} \pm 0.10 \times 10^{-5}$	$3.37 \times 10^{-5} \pm 0.16 \times 10^{-5}$	-	-	$2.95 \times 10^{-5} \pm 0.10 \times 10^{-5}$
k_2^*	-	$6.16 \times 10^{-2} \pm 0.84 \times 10^{-2}$	-	-	-
k_1	$5.50 \times 10^{-2} \pm 0.49 \times 10^{-2}$	-	-	-	-
K_{D1}	-	-	-	-	$5.11 \times 10^{-2} \pm 0.61 \times 10^{-2}$
K_{D2}	-	-	-	-	-
k_w	-	-	-	$5.12 \times 10^{-6} \pm 0.80 \times 10^{-6}$	-
n_w	-	-	-	0.37 ± 0.04	-
D1	-	-	-	-	$2.86 \times 10^{-5} \pm 0.12 \times 10^{-5}$
D2	-	-	-	-	-
k_{ipd}	-	-	$2.52 \times 10^{-6} \pm 0.26 \times 10^{-6}$	-	-
c	-	-	$2.72 \times 10^{-6} \pm 1.39 \times 10^{-6}$	-	-
chi-sq	8.78×10^{-11}	1.16×10^{-10}	4.51×10^{-10}	7.91×10^{-7}	8.35×10^{-11}

Table S8. Fitting parameters and chi square (chi-sq) values for K1, K2, IPD, Weibull and DE equations applied to the uptake of Cu(II) ions by the pHEMA/PVP semi-IPN at pH 8. In this case, only the reduced DE equation (i.e. single exponential) provided good fits of the experimental data.

	K1	K2	IPD	Weibull	DE
q_e	$2.31 \times 10^{-4} \pm 0.05 \times 10^{-4}$	$3.21 \times 10^{-4} \pm 0.12 \times 10^{-4}$	-	-	$2.32 \times 10^{-4} \pm 0.05 \times 10^{-4}$
k_2^*	-	$1.31 \times 10^{-2} \pm 0.01 \times 10^{-2}$	-	-	-

k_1	$1.64 \times 10^{-2} \pm 0.07 \times 10^{-2}$	-	-	-	-
K_{D1}	-	-	-	-	$1.64 \times 10^{-2} \pm 0.0931 \times 10^{-2}$
K_{D2}	-	-	-	-	-
k_w	-	-	-	$1.05 \times 10^{-5} \pm 0.160 \times 10^{-5}$	-
n_w	-	-	-	0.61 ± 0.03	-
D1	-	-	-	-	$2.31 \times 10^{-4} \pm 0.05 \times 10^{-4}$
D2	-	-	-	-	-
k_{ipd}	-	-	$1.66 \times 10^{-5} \pm 0.10 \times 10^{-5}$	-	-
c	-	-	$-7.28 \times 10^{-22} \pm 5.23 \times 10^{-6}$	-	-
chi-sq	5.44×10^{-10}	3.54×10^{-10}	5.17×10^{-9}	3.05×10^{-9}	3.54×10^{-10}

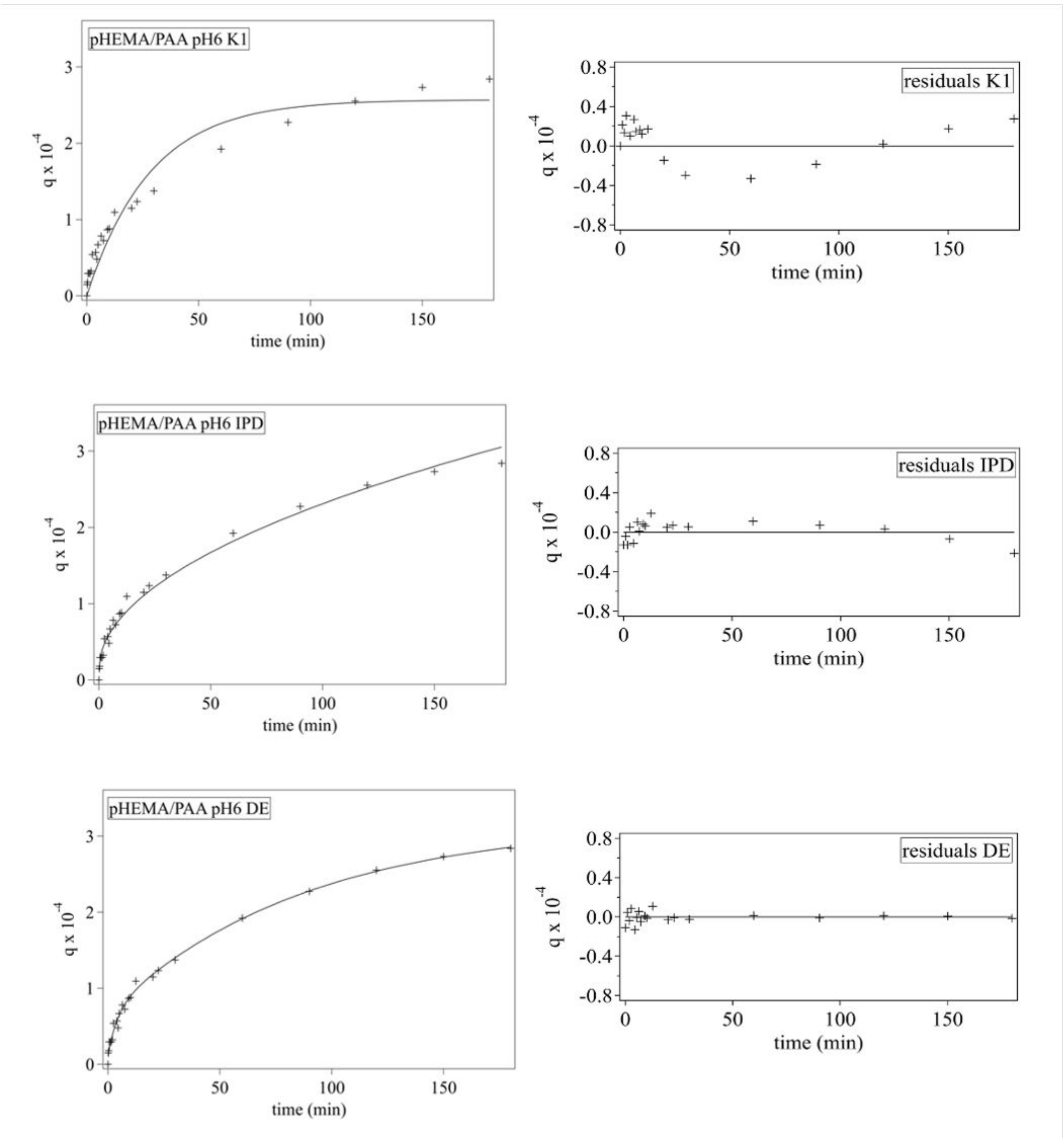

Figure S7. Fitting of q (grams of solute sorbed per gram of sorbent) and residuals over time for the uptake of Cu(II) ions by the pHEMA/PAA semi-IPNs at pH 6 using K1, IPD and DE equation. See main text for K2 and Weibull fitting of q and residuals over time.

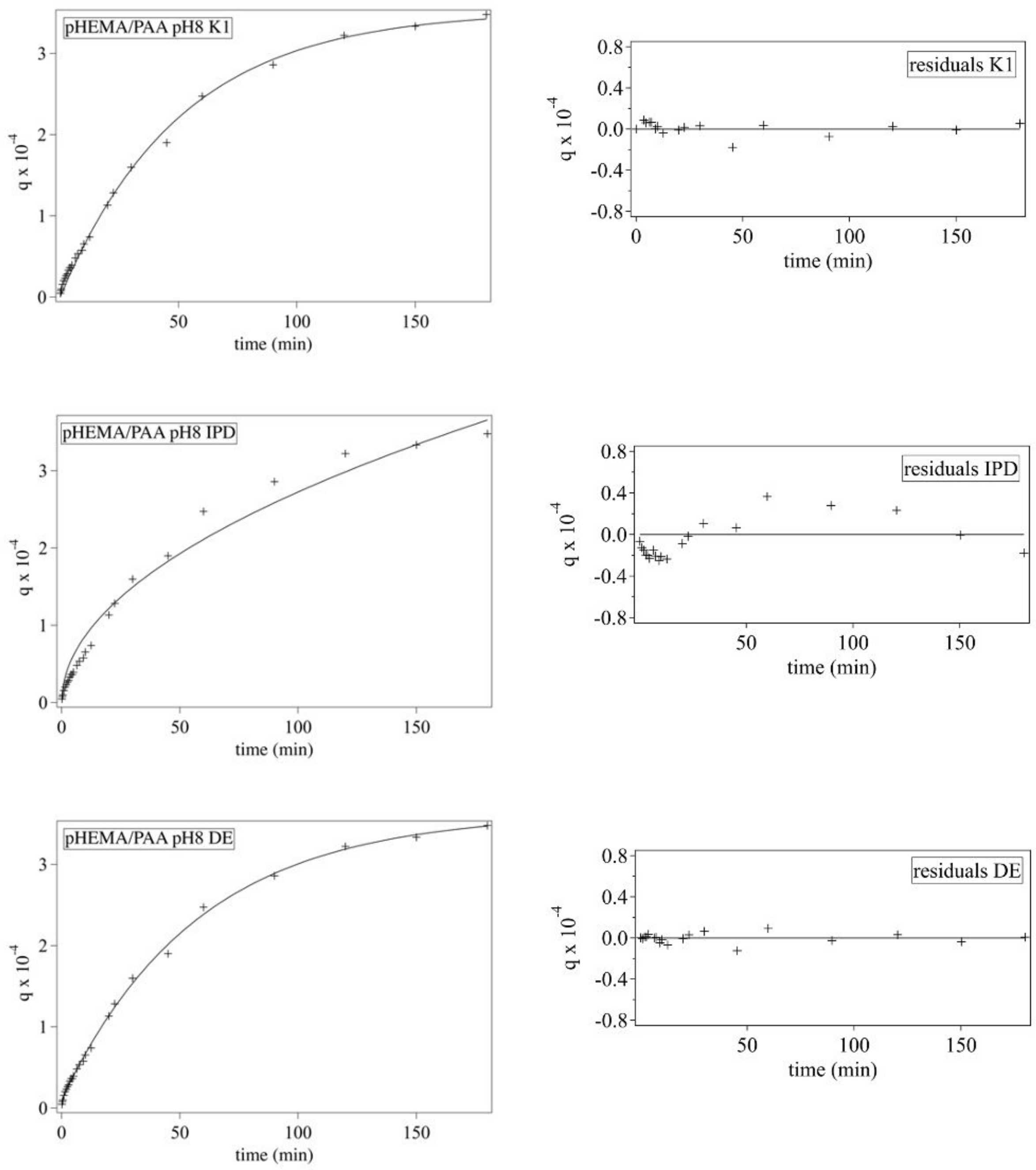

Figure S8. Fitting of q (grams of solute sorbed per gram of sorbent) and residuals over time for the uptake of Cu(II) ions by the pHEMA/PAA semi-IPNs at pH 8 using K1, IPD and DE equation. See main text for K2 and Weibull fitting of q and residuals over time.

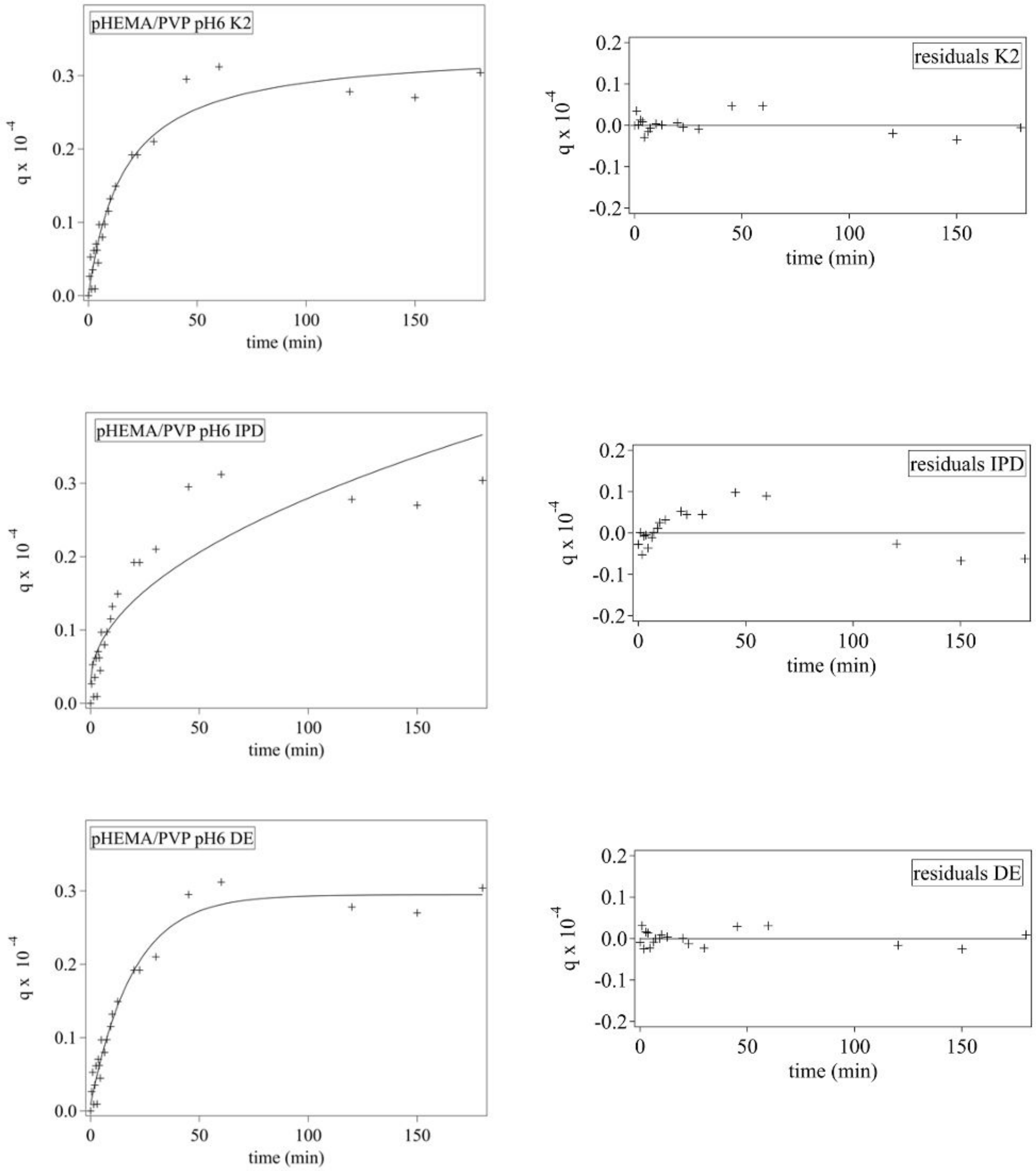
pHEMA/PVP pH6 K2
residuals K2
pHEMA/PVP pH6 IPD
residuals IPD
pHEMA/PVP pH6 DE
residuals DE
q x 10^-4
time (min)

Figure S9. Fitting of q (grams of solute sorbed per gram of sorbent) over time for the uptake of Cu(II) ions by the pHEMA/PVP semi-IPNs at pH 6 using K2, IPD and DE equation. See main text for K1 and Weibull fitting of q and residuals over time.

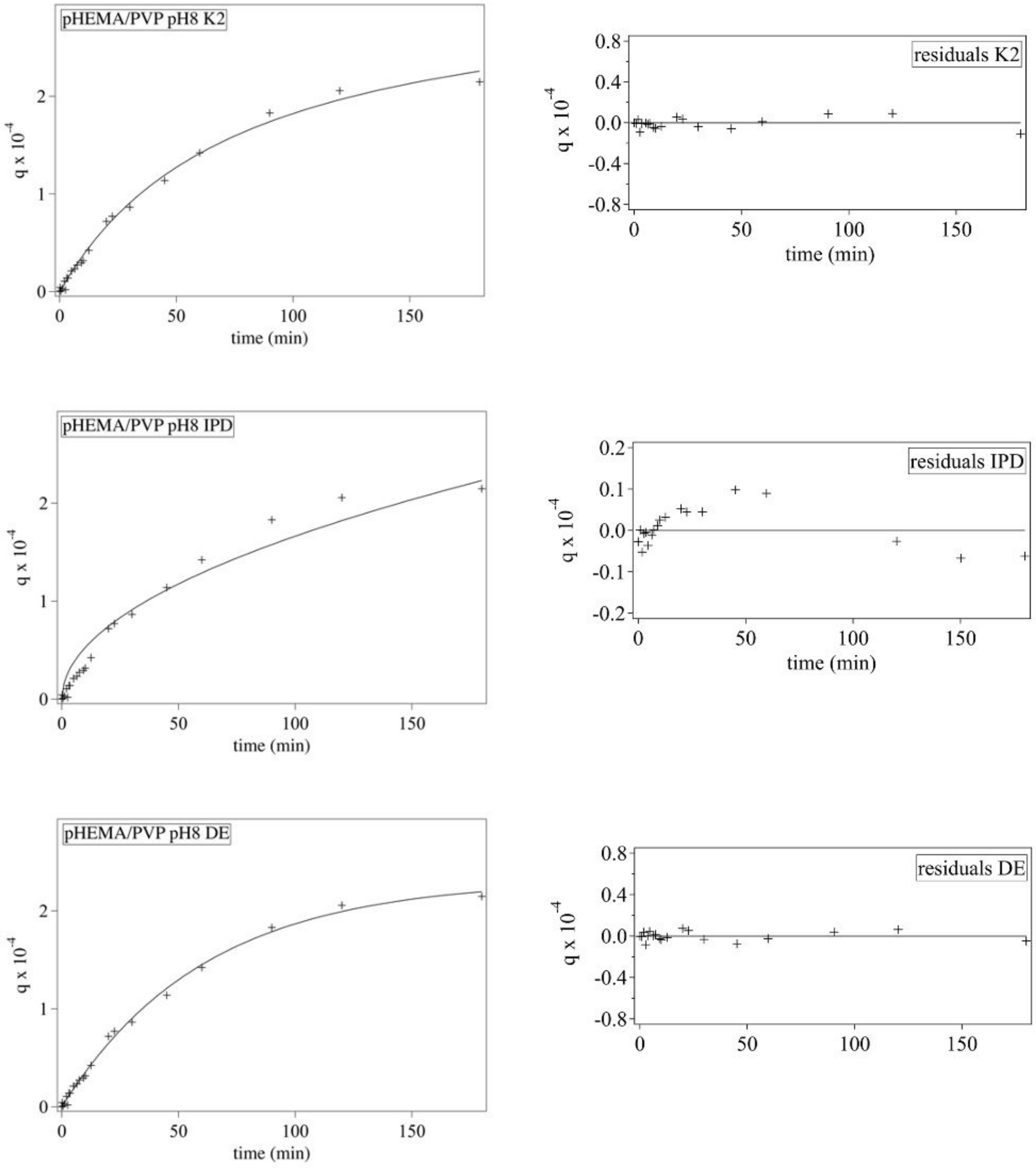
pHEMA/PVP pH8 K2
q x 10^-4
time (min)
residuals K2
pHEMA/PVP pH8 IPD
residuals IPD
pHEMA/PVP pH8 DE
residuals DE

Figure S10. Fitting of *q* (grams of solute sorbed per gram of sorbent) over time for the uptake of Cu(II) ions by the pHEMA/PVP semi-IPNs at pH 8 using K2, IPD and DE equation. See main text for K1 and Weibull fitting of *q* and residuals over time.

References:

[1] Noferini, D.; Faraone, A.; Rossi, M.; Mamontov, E.; Fratini, E.; Baglioni, P. Disentangling Polymer Network and Hydration Water Dynamics in Polyhydroxyethyl Methacrylate Physical and Chemical Hydrogels. *J. Phys. Chem. C* **2019**, *123* (31), 19183-19194.

[2] Okay, O. Macroporous Copolymer Networks, in Progress in Polymer Science , Oxford, **2000**.

[3] Lagergren, S. Zur theorie der sogenannten adsorption gelöster Stoffe. Stockholm Kongl. svenska vetenskaps-akad. *Handlinger* **1898**, *24*, 1-39. doi: 10.4236/ss.2014.52008.

[4] Ho, Y.S.; McKay; G. Pseudo-second order model for sorption processes. *Process Biochem.*, **1999**, *34*, 451-465. doi: 10.1016/S0032-9592(98)00112-5.

[5] Aharoni, C; Sparks, D. *Kinetics of soil chemical reactions - a theoretical treatment*, in Rates of Soil Chemical Processes, Madison, WI, Sparks, D. L. and Suarez, D. L., Eds. Soil Science Society of America, **1991**, 1-18.

[6] Robati, D. Pseudo-second-order kinetic equations for modeling adsorption systems for removal of lead ions using multi-walled carbon nanotube, *J. Nanostructure Chem.* **2013**, *3* (55) doi: 10.1186/2193-8865-3-55.

[7] Papadopoulou, V.; Kosmidis, K.; Vlachou, M.; Macheras, P. On the use of the Weibull function for the discernment of drug release mechanisms, *Int. J. of Pharm.* **2006**, *309* (1-2), 44-50.

[8] Wilczak, A.; Keinath, T.M. Kinetics of sorption and desorption of copper(II) and lead(II) on activated carbon. *Water Environ. Res.* **1993**, *65* (3), 238-44.
